# GABA_A_ receptors and neuroligin 2 synergize to promote synaptic adhesion and inhibitory synaptogenesis

**DOI:** 10.3389/fncel.2024.1423471

**Published:** 2024-07-18

**Authors:** Yusheng Sui, Martin Mortensen, Banghao Yuan, Martin W. Nicholson, Trevor G. Smart, Jasmina N. Jovanovic

**Affiliations:** ^1^Department of Pharmacology, School of Pharmacy, University College London, London, United Kingdom; ^2^Department of Neuroscience, Physiology and Pharmacology, Division of Biosciences, University College London, London, United Kingdom

**Keywords:** inhibition, GABAergic synapse, synaptic, extrasynaptic, stable cell lines, medium spiny neurons, immunocytochemistry, protein domains

## Abstract

GABA_A_ receptors (γ-aminobutyric acid-gated receptors type A; GABA_A_Rs), the major structural and functional postsynaptic components of inhibitory synapses in the mammalian brain, belong to a family of GABA-gated Cl^−^/HCO_3_^−^ ion channels. They are assembled as heteropentamers from a family of subunits including: α (1–6), β(1–3), γ(1–3), δ, ε, π, θ and ρ(1–3). GABA_A_Rs together with the postsynaptic adhesion protein Neuroligin 2 (NL2) and many other pre- and post-synaptic proteins guide the initiation and functional maturation of inhibitory GABAergic synapses. This study examined how GABA_A_Rs and NL2 interact with each other to initiate the formation of synapses. Two functionally distinct GABA_A_R subtypes, the synaptic type α2β2γ2-GABA_A_Rs versus extrasynaptic type α4β3δ-GABA_A_Rs were expressed in HEK293 cells alone or together with NL2 and co-cultured with striatal GABAergic medium spiny neurons to enable innervation of HEK293 cells by GABAergic axons. When expressed alone, only the synaptic α2β2γ2-GABA_A_Rs induced innervation of HEK293 cells. However, when GABA_A_Rs were co-expressed with NL2, the effect on synapse formation exceeded the individual effects of these proteins indicating a synergistic interaction, with α2β2γ2-GABA_A_R/NL2 showing a significantly greater synaptogenic activity than α4β3δ-GABA_A_R/NL2 or NL2 alone. To investigate the molecular basis of this interaction, different combinations of GABA_A_R subunits and NL2 were co-expressed, and the degree of innervation and synaptic activity assessed, revealing a key role of the γ2 subunit. In biochemical assays, the interaction between NL2 and α2β2γ2-GABA_A_R was established and mapped to the large intracellular domain of the γ2 subunit.

## 1 Introduction

GABA_A_ receptors are the essential structural and functional postsynaptic components of inhibitory synapses in the mammalian brain. They belong to a diverse family of GABA-gated Cl^−^/HCO_3_^−^ permeable ion channels that promote neuronal differentiation and synaptogenesis in the developing brain by increasing neuronal excitability (Owens and Kriegstein, 2002; Raimondo et al., 2017; Cherubini and Ben-Ari, 2023). In contrast, in the adult brain, GABA_A_Rs mediate inhibitory neurotransmission by decreasing neuronal excitability in a process that is fundamental to normal brain function and information processing (Schofield et al., 1987; Olsen and Sieghart, 2009; Smart and Stephenson, 2019; Sallard et al., 2021).

GABA_A_Rs are hetero-pentameric assemblies of subunits selected from: *α* (1–6), *β* (1–3), *γ* (1–3), δ, ε, π, θ and ρ(1–3), in which two α, two β and one γ subunit are required for the formation of fully functional synaptic receptors (Olsen and Sieghart, 2009; Chua and Chebib, 2017; Scott and Aricescu, 2019). The subtypes of GABA_A_Rs which incorporate α1-3 and 5, β2-3 and γ2 subunits are spatially, functionally, and pharmacologically distinct from those containing the α4, 6, β2-3 and δ subunits (Farrant and Nusser, 2005; Smart and Mortensen, 2023). The presence of the γ2 subunit is obligatory for synaptic GABA_A_Rs because it governs their localization and clustering at the postsynaptic membrane, allowing for the rapid and robust neurotransmission in all GABAergic synapses (Olsen and Sieghart, 2009; Lorenz-Guertin et al., 2018). However, the γ2-containing GABA_A_Rs translocate in and out of synapses as part of their lifecycle. Thus, they are not solely a synaptic feature, but do also transit through the extrasynaptic space (Thomas et al., 2005; Hannan et al., 2020). In contrast, the subtypes of GABA_A_Rs lacking the γ2 subunit (αβ isoforms), or those incorporating the δ subunit are predominantly located outside of synapses where they can be activated by low levels of ambient GABA to mediate tonic inhibition (Farrant and Nusser, 2005; Smart and Mortensen, 2023). While the incorporation of the β subunit is required for the expression of GABA_A_Rs at the neuronal cell surface (Connolly et al., 1996a,b; Nguyen and Nicoll, 2018), some α subunits are selectively assembled at specific inhibitory synapses where they support the formation and function of neuronal circuits involved in specific brain physiology, such as anxiety, sedation, arousal, and others (Klausberger et al., 2002; Rudolph and Mohler, 2006; Thomson and Jovanovic, 2010).

How, where, and when inhibitory synapses are formed is tightly regulated during brain development by genetic and environmental factors and guided by specialized protein–protein interactions leading to the formation of transsynaptic complexes between the pre-and postsynaptic elements. Multiple proteins have been shown to participate in the initiation and functional maturation of inhibitory synapses, including the postsynaptic adhesion protein NL2 together with its presynaptic partners α and β neurexins (Sudhof, 2017; Sudhof, 2018; Ali et al., 2020), as well as other synaptic partners, such as SLITRK3, β-dystroglycan, IgSF9b, GARLH4/LHFPL4 (Connor and Siddiqui, 2023). GABA_A_Rs themselves participate in inhibitory synaptogenesis as structural (Fuchs et al., 2013; Brown et al., 2014, 2016; Duan et al., 2019) and signaling (Pallotto and Deprez, 2014; Arama et al., 2015; Cherubini and Ben-Ari, 2023) components and contribute to the functional specialization of synapses via the incorporation of specific receptor subtypes with distinct physiological properties (Thomson and Jovanovic, 2010; Fritschy et al., 2012; Fritschy and Panzanelli, 2014).

Constitutive and inducible gene knockout studies of individual proteins involved in synapse formation in mice, including NL2, have revealed subtle deficits in inhibitory synapses, without causing a global impairment of GABAergic synaptogenesis (Fritschy et al., 2012; Sudhof, 2018). This suggests that the process of synapse initiation and functional maturation relies on multiple protein complexes and, importantly, their specific interactions which incrementally and cooperatively contribute to this process. While molecular details of these interactions remain largely unknown, their importance has been demonstrated in the developing hippocampus where cooperative interaction between NL2 and SLITRK3 is required for the formation and functional maturation of inhibitory synapses (Li et al., 2017).

Our initial studies have demonstrated a cooperative interaction between GABA_A_Rs and NL2 in promoting the formation and strengthening of synaptic connections in a co-culture model in which HEK293 cells expressing GABA_A_Rs and NL2 were cultured together with GABAergic medium spiny neurons (Fuchs et al., 2013). Here, we have explored this cooperativity further to uncover the molecular details of the GABA_A_R/NL2 interaction. Our experiments demonstrate that the prototypical synaptic α2β2γ2-GABA_A_Rs have a significantly greater effect in facilitating the NL2-dependent induction of synapses than the prototypical extrasynaptic α4β3δ-GABA_A_Rs. Furthermore, we demonstrate that the synergism between GABA_A_Rs and NL2 is mediated by the γ2 subunit interaction with NL2, and we map this interaction to the intracellular domain of this subunit.

## 2 Materials and methods

### 2.1 Cell lines, primary neuronal cultures and co-cultures

Human embryonic kidney cells (ATCC) were maintained using Dulbecco’s Minimum Essential Medium (DMEM; Thermo Fischer) supplemented with 10% v/v fetal bovine serum (FBS; 10082–147, Thermo Fischer), 10 mM L-Glutamine (25030–024, Thermo Fischer), 50 units/mL penicillin and 50 μg/mL streptomycin (15140–148, Thermo Fischer). HEK293 cell lines stably expressing GABA_A_Rs were kept in complete DMEM with the addition of 800 μg/mL Geneticin G418 sulfate (11811023, Thermo Fischer), 800 μg/mL Zeocin (R25001, Gibco), and 800 μg/mL hygromycin B (10687010, Invitrogen) for selection of α2 and α4 subunits, β2 and β3 subunits, and γ2 and δ subunits, respectively. The α2β2γ2-GABA_A_R stable cell line was described previously (Brown et al., 2014) and in Supplementary Figure S1.

The GABAergic medium spiny neuron cultures were prepared from the striatum of ~ day E17 embryonic Sprague–Dawley rats (UCL-BSU) housed and sacrificed according to United Kingdom Home Office guidelines as previously described (Brown et al., 2014). Briefly, brains were dissected in sterile Ca^2+^ and Mg^2+^ − free HEPES-buffered saline solution (HBSS; 14180–046, Thermo Fischer) to obtain striata. Neurons were dissociated using fire-polished Pasteur glass pipettes, counted using a hemocytometer, and plated onto poly-D-lysine (0.1 mg/mL, P1149-10MG, Sigma Aldrich) coated tissue culture dishes (Z707686, TPP) for biochemical experiments, or poly-L-lysine (0.1 mg/mL, P6282-5MG, Sigma Aldrich) coated 13 mm glass coverslips (631-0148P, VWR) for immunolabeling and electrophysiology. Neuronal cultures and co-cultures with HEK293 cells were maintained in Neurobasal medium (21103–049, Gibco) containing B27 supplement (17504–044, Gibco), glutamine (2 mM, 25030–024, Gibco), penicillin (50 units/mL, 15070–63, Gibco), streptomycin (50 g/mL, 15070–063, Gibco), and glucose (6 mM, G8769, Sigma).

In order to generate a HEK293 neuronal co-culture, adherent HEK293 cells (control or stably expressing GABA_A_Rs) were first transfected with the cDNA of proteins of interest using Effectene (301425, Qiagen), and 24 h later these cells were transferred to the medium spiny neuron culture for a further 24–48 h of incubation. as described previously (Brown et al., 2014).

### 2.2 Construction of HEK293 cell line stably expressing α4β3δ-GABA_A_Rs

GABA_A_R α4, β3 and δ cDNAs were cloned into pcDNA3-G418 (Invitrogen), pcDNA3.1-zeocin (Invitrogen) and pcDNA3.1-hygromycin (Invitrogen), respectively, for antibiotic-selective expression. Lipofectamine LTX (15338–030, Invitrogen) was used for the two-stage stable transfection. For the first stage, α4 and β3 cDNAs were transfected into HEK293 cells followed by G418 and zeocin antibiotic selection. For the second stage, the HEK293 cell line clone expressing both subunits was transfected with δ cDNA followed by G418, zeocin, and hygromycin antibiotic selection. Stable expression of the subunits was confirmed by immunoblotting and immunolabeling using subunit-specific antibodies and their functional properties were assessed using whole-cell recordings (Supplementary Figure S2).

### 2.3 Cell surface ELISA

HEK293 cells stably expressing GABA_A_Rs were transfected with HA-tagged NL2 cDNA (Poulopoulos et al., 2009) using Effectene (301,425, Qiagen), and incubated in 24-well plates coated with 0.1 mg/mL poly-D-lysine (P1149, Sigma Aldrich) in the 37°C 5% CO2 humidified incubator for 24 h. The cells were fixed with 4% paraformaldehyde (PFA)/4% sucrose w/v in PBS for 10 min at room temperature and subsequently washed with PBS and HBSS (14185–052, Gibco). Cells were blocked in 1% bovine serum albumin (BSA, BP9704-100, Fisher Scientific) in HBSS for 30 min at room temperature and subsequently incubated with anti-HA tag primary antibody (1,10,000 in blocking solution, ab184643, Abcam) for 2 h at room temperature or overnight at 4°C. For assessing the total expression of proteins, cells were permeabilized with 0.5% Triton X-100 (A16046, Alfa-Aesar) in HBSS for 10 min at room temperature before the blocking step. After incubation, cells were washed, blocked in 1% BSA in HBSS for 30 min at room temperature, and incubated with the secondary antibody conjugated to horseradish peroxide (HRP) (31,460, Thermo Fisher) in 1% BSA/ HBSS (1,2,500) for 1 h at room temperature. The cells were washed with HBSS and the HRP activity was detected using tetramethylbenzidine reagent (TMB, 34028, Thermo Scientific). The oxidation of TMB produced blue color, the absorbance of which was measured at 650 nm by DU800 spectrophotometer (Beckman Coulter).

### 2.4 Immunocytochemistry

The cells plated on 13 mm coverslips coated with poly-L-lysine were briefly washed with PBS and fixed with 4% PFA/4% sucrose in PBS for 10 min at room temperature. For assessing the activity of the presynaptic terminals, Cy5-labeled anti-synaptotagmin 1 luminal domain-specific antibody (1,50, 105311C5, Synaptic Systems) was added to the culture and incubated in the 37°C 5% CO2 humidified incubator for 30 min before fixation. The PFA was aspirated, and the cells were washed thoroughly with PBS. The residual aldehyde groups of PFA were blocked with 0.3 M glycine in PBS for 10 min at room temperature, followed by multiple washes with PBS. The cells were blocked in 1% BSA in PBS for 30 min at room temperature. The primary antibodies: anti-VGAT (1,500, 131,003, Synaptic Systems), anti-GABA_A_R α2 subunit (1,500, 224,103, Synaptic Systems), anti-GABA_A_R α4 subunit (1,200, Hörtnagl et al., 2013), anti-GABA_A_R β2/3 subunit (1,500, MAB341, Sigma Aldrich), anti-GABA_A_R γ2 subunit (1,2,500, Fritschy and Mohler, 1995), anti-GABA_A_R δ subunit (1,200, 868-GDN, PhosphoSolutions), and anti-Bassoon (1,500, MA1-20689, Thermo Fischer) were diluted in 1% BSA in PBS, added to the cells, and incubated for 2 h at room temperature or overnight at 4°C. After incubation, the cells were rinsed twice and washed multiple times with PBS. For labeling of intracellular proteins, the cells were permeabilized with 0.5% Triton-X-100 in PBS for 10 min at room temperature prior to the addition of the primary antibody mix. The cells were washed and blocked again with 1% BSA in PBS for 30 min at room temperature. Fluorescently-labeled secondary antibodies (AlexaFluor, Invitrogen) were diluted in 1% BSA in PBS (1,750) and added to the cells for 1 h at room temperature protected from light. The coverslips were washed with PBS and mounted on glass slides with ProLong Gold antifade reagent (P36930, Invitrogen). The slides were dried at room temperature protected from light and kept at 4°C in boxes.

### 2.5 Confocal imaging and analysis

The coverslips were imaged using a Zeiss confocal microscope LSM 700, 710, or 880 with 63× oil immersion objective and analyzed using ImageJ (National Institute of Health) as described previously (Brown et al., 2016). Images were acquired at 12-bit depth from 10 to 15 cells from each co-culture. For each image, a series of z-stack images were acquired from the bottom to the top of HEK293 cells with optimal intervals of 0.7 μm. ImageJ software was utilized for the analysis of contacts formed between presynaptic GABAergic terminals of cultured neurons and HEK293 cells. The co-localization was obtained by the *Process → Image Calculator* function using the option *and* which produced an image showing all the pixels that appeared in both channels. The threshold of the image was adjusted by the *Auto-Threshold* function. The data for co-localization were obtained by the *Analyze → Analyze Particles* function. For quantitative assessment of synaptic contacts formed between presynaptic GABAergic terminals and HEK293 cells, the *% area* was selected because this parameter represents the surface area of each HEK293 cell with co-localized pixels normalized to the total surface area of the cell, which therefore accounts for the difference in size of individual HEK293 cells. These parameters were imported into Origin Pro software for statistical analysis and graphical presentation of the data. The data were plotted with Box-and-Whisker plots showing the median, standard deviation, and outliers. The normal (Gaussian) distribution of the data was first tested using the Shapiro–Wilk normality test. Non-normally distributed data were subjected to non-parametric Mann–Whitney test or Kruskal Wallis ANOVA followed by Dunn’s test for multiple comparisons. Normally distributed data were analyzed using a two-tailed Student’s t-test to determine the statistical significance.

### 2.6 Super-resolution imaging and analysis

The samples were prepared in the same way as for the confocal imaging. The GABA_A_Rs were labeled with α2 (1,500, 224,103, Synaptic Systems) or α4 (1,200, Hörtnagl et al., 2013) subunit-specific antibodies, respectively. The synaptic contacts were labeled with presynaptic active zone marker Bassoon-specific antibody (MA1-20689, Thermo Fischer). Images were acquired using ELYRA PS.1 SIM (Carl Zeiss) at 63x oil lens following chromatic shift correction by recording fluorescent beads. A 4 μm z-stack with 0.110 μm intervals of the samples was acquired to keep the z-range in focus. The images were then processed by the Structured Illumination and Channel Alignment function in Zen 2012 Software. The images were deconvolved and analyzed using SVI Huygens Professional software. After deconvolution, the background was eliminated using the Costes Optimized method (Coastes et al., 2004). The z-stack images were rendered to 3-dimensional images for colocalization analysis and Manders coefficients were calculated to show the level of overlapping signals, with M1 indicating the proportion of Bassoon overlapping with GABA_A_Rs and M2 indicating the proportion of GABA_A_Rs overlapping with Bassoon.

### 2.7 Immunoblotting

Cultured cells were lysed with 2% SDS and the protein concentration determined using the BCA assay (Thermo Fisher Scientifics). Proteins were separated on 10% SDS-poly-acrylamide separation gels and transferred onto a solid nitrocellulose membrane (Whatman). For the detection of proteins, the following primary antibodies were used: anti-HA tag (1,1,000, ab184643, Abcam), anti-GABA_A_R α1 subunit (1,500, Duggan and Stephenson, 1990), anti GABA_A_R β3 subunit (1:200, UCL 74, (Tretter et al., 1997)), anti-GABA_A_R δ subunit (1,200, 868-GDN, PhosphoSolutions) and anti NL2 (1,800, 129,203, Synaptic Systems). Anti-alkaline phosphatase-conjugated secondary antibodies: anti-rabbit (1,1,000, A16099, Invitrogen) and anti-mouse (1,200, 31,450, Invitrogen) were used for visualization of the protein bands.

### 2.8 Co-immunoprecipitation

Protein lysates were obtained from adult male rat cortex or HEK293 cells expressing GABA_A_Rs/NL2 via homogenization in lysis buffer (20 mM HEPES pH 7.4, 100 mM NaCl, 1% Triton X-100, 2 mM CaCl_2_, 1 mM MgCl_2_), containing phenylmethylsulfonyl fluoride (PMSF;10 μM), leupeptin, chymostatin, pepstatin (5 μg/mL each, Peptide Institute). The concentration of the protein lysates was determined by Bradford assay (Bio-Rad). The input (1 mg total protein) was incubated with 10 μg of GABA_A_R α1 or α2 subunit-specific antibodies (Duggan and Stephenson, 1990), or anti-HA tag (ab184643, Abcam) or anti-c-myc antibody (2 μg/mL, 05–724, Millipore) or non-immune control antibodies (31,243, Invitrogen, from the same species as the specific antibody) followed by 1% BSA coated Protein-G-Sepharose beads (50 μL, NB-45-00037-5, Generon). The beads were pulled down by centrifugation after extensive washing and denatured with Laemmli SB (62.5 mM Tris, pH 8.0, 2% SDS, 10% glycerol, 0.0025% Bromophenol Blue, 100 mM DTT) for SDS/PAGE and immunoblotting.

### 2.9 Electrophysiology

Coverslips with cells (transfected HEK293, stable HEK293 cell lines, neurons or HEK293/neuron co-cultures) were transferred into a recording chamber on a Nikon Eclipse FN1 microscope, where cells were continuously perfused with Krebs solution containing (mM): 140 NaCl, 4.7 KCl, 1.2 MgCl_2_, 2.52 CaCl_2_, 11 Glucose and 5 HEPES (pH 7.4). Patch pipettes (thin-walled filamented borosilicate glass capillaries; TW150F-4; WPI, United States; 3–4 MΩ) were filled with an intracellular solution containing (mM): 140 CsCl, 2 NaCl, 2 MgCl_2_, 5 EGTA, 10 HEPES, 0.5 CaCl_2_, 2 Na-ATP and 0.5 Na-GTP (pH 7.2). To record functional current GABA_A_R responses, drugs were applied to cells using a Y-tube delivery system (Mortensen and Smart, 2007).

HEK293 cells were voltage-clamped at −40 mV using an Axopatch 200B amplifier (Molecular Devices, United States), and whole-cell currents (IPSCs or drug-activated) were filtered at 5 kHz (−36 dB), digitized at 50 kHz via a Digidata 1322A (Molecular Devices), and recorded to a Dell Optiplex 990 using Clampex 10.2 (Molecular Devices). Series resistance was compensated at 70%, and only data with less than 20% deviation in series resistance was included in subsequent analyses.

## 3 Results

### 3.1 Synergistic interaction between GABA_A_Rs and NL2 in synapse formation

**Figure 1:**
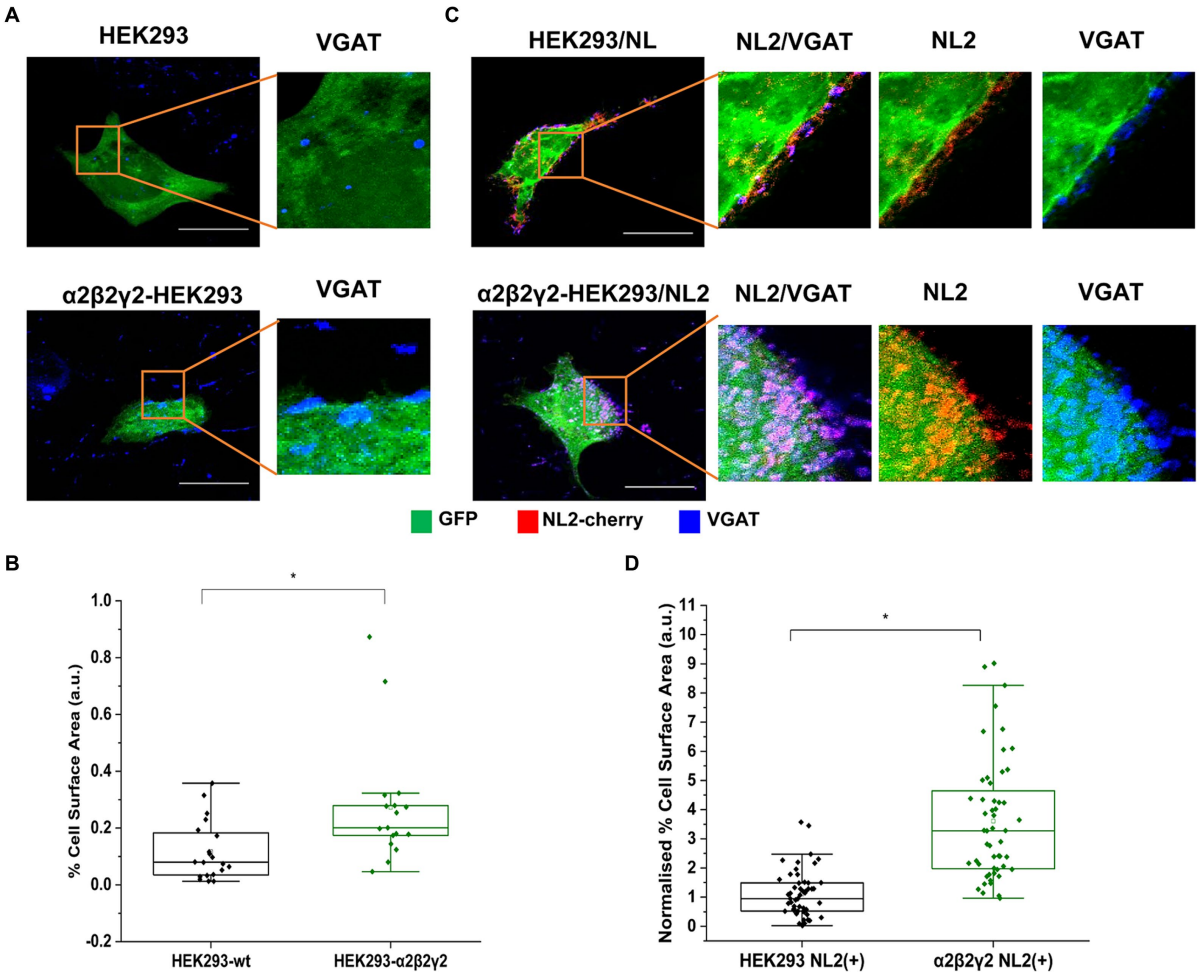
α2β2γ2-GABA_A_Rs induce synaptic contact formation and potentiate the induction of contacts by NL-2. Synaptic contact formation in co-culture of embryonic medium spiny neurons and **(A)** HEK293 (wt; upper panels) or α2β2γ2-GABA_A_R-expressing HEK293 cells (lower panels), or **(C)** HEK293/NL2 cells (wt; upper panels) or α2β2γ2-GABA_A_R/NL2-expressing cells (lower panels). The HEK293 cell body was visualized by GFP (green), GABAergic terminals were labeled with an anti-VGAT antibody (blue) and NL2 was mcherry-tagged (red). Scale bar = 20 μm. Fluorescent imaging was done using Zeiss 700 confocal microscope at 63 × magnification with image size 1,024 × 1,024. Max intensity projection of the z-stack images was shown. The enlarged images are 10 × zoom in. Quantitative analysis of synapses expressed as % area of co-localized pixels that represent contacts between VGAT terminals and in **(B)** HEK293 or α2β2γ2-GABA_A_R-expressing cells (*n* = 21 and *n* = 18, respectively; *N* = 2 independent experiments, *p* = 0.002), or **(D)** HEK293/NL2 cells or α2β2γ2-GABA_A_R/NL2 expressing cells (n = 53 and n = 52 cells, respectively; *N* = 3 independent experiments, *p* < 0.00001 (4.7 × 10^−13^)). The box and whisker plot show the mean (square dot with no fill), median (horizontal line), and standard deviation (whiskers). Shapiro–Wilk normality test was used to test the normal distribution of the data and Mann–Whitney test was used to analyze statistical significance of the difference (*p < 0.05).

We have demonstrated previously that stable expression of synaptic α1β2γ2-GABA_A_Rs in HEK293 cells promotes the adhesion of GABAergic axons and the formation of functional synapses when these cells are co-cultured with the GABAergic medium spiny neurons (Fuchs et al., 2013; Brown et al., 2014, 2016). When GABA_A_Rs were co-expressed with NL2 in this system, the effect on synapse formation exceeded the individual effects of these two proteins both in the number and transmission efficacy of the synapses ascertained by electrophysiological recordings (Fuchs et al., 2013). To investigate the degree of HEK293 cell innervation induced by another synaptic GABA_A_R type and possible synergistic interaction with NL2, in the current study, we have expressed and functionally characterized the HEK293 stable cell line expressing the α2β2γ2-GABA_A_R (Supplementary Figures S1A,B; Brown et al., 2014). These cells were fluorescently labeled by transiently expressing the green fluorescent protein (GFP) and co-cultured with GABAergic medium spiny neurons (Figure 1A). The control HEK293 cells were also labeled with GFP. The cells were fixed and immunolabeled with a VGAT-specific antibody to detect GABAergic terminals forming contacts with HEK293 cells using confocal microscopy. Synaptic contacts were defined based on signal colocalization between the VGAT and GFP (green and blue channels in Figure 1A) and analyzed using Image J as described in the Methods and previously (Brown et al., 2014). Quantification of the % area of co-localized pixels that represent contacts between VGAT terminals and HEK293 cells revealed a significant increase in synaptic contact formation in the presence of α2β2γ2-GABA_A_Rs in comparison with the control HEK293 cell line (median = 0.20%; IQR = 0.16 - 0.30%; *n* = 17 cells vs. median = 0.08%; IQR = 0.03 - 0.21%; *n* = 21 cells, respectively; from *N* = 2 independent experiments, *p* = 0.002, Figure 1B).

To investigate how the co-expression of GABA_A_Rs and NL2 in HEK293 cells may regulate the formation of synaptic contacts, HEK293 cells stably expressing α2β2γ2-GABA_A_Rs or the control HEK293 cells were transfected with GFP and cherry-tagged NL2 cDNAs, and co-cultured with the medium spiny neurons (Figure 1C). Expression of NL2 in control HEK293 cells induced synaptic contact formation in agreement with the published literature [median = 0.95%; IQR = 0.52 - 1.49%; *n* = 53 cells from *N* = 3 independent experiments; *p* < 0.00001 (*p* = 3.5 × 10^−9^)]; (Scheiffele et al., 2000) but when NL2 was expressed in the α2β2γ2-GABA_A_R stable cell line, the formation of contacts was significantly greater [median = 3.28%; IQR = 1.96 - 4.78%; *n* = 52 cells from *N* = 3 independent experiments; *p* = < 0.00001 (*p* = 4.7 × 10^−13^); Figure 1D]. In this analysis, the value of the % area of co-localized pixels for each HEK293 cell was divided by the fluorescence value of NL2 measured in the same cell because of the high degree of variation in expression of NL2 following transient transfection. These results support the previously described (Fuchs et al., 2013) strong synergistic interaction between GABA_A_Rs and NL2 during the formation of synaptic contacts.

**Figure 2:**
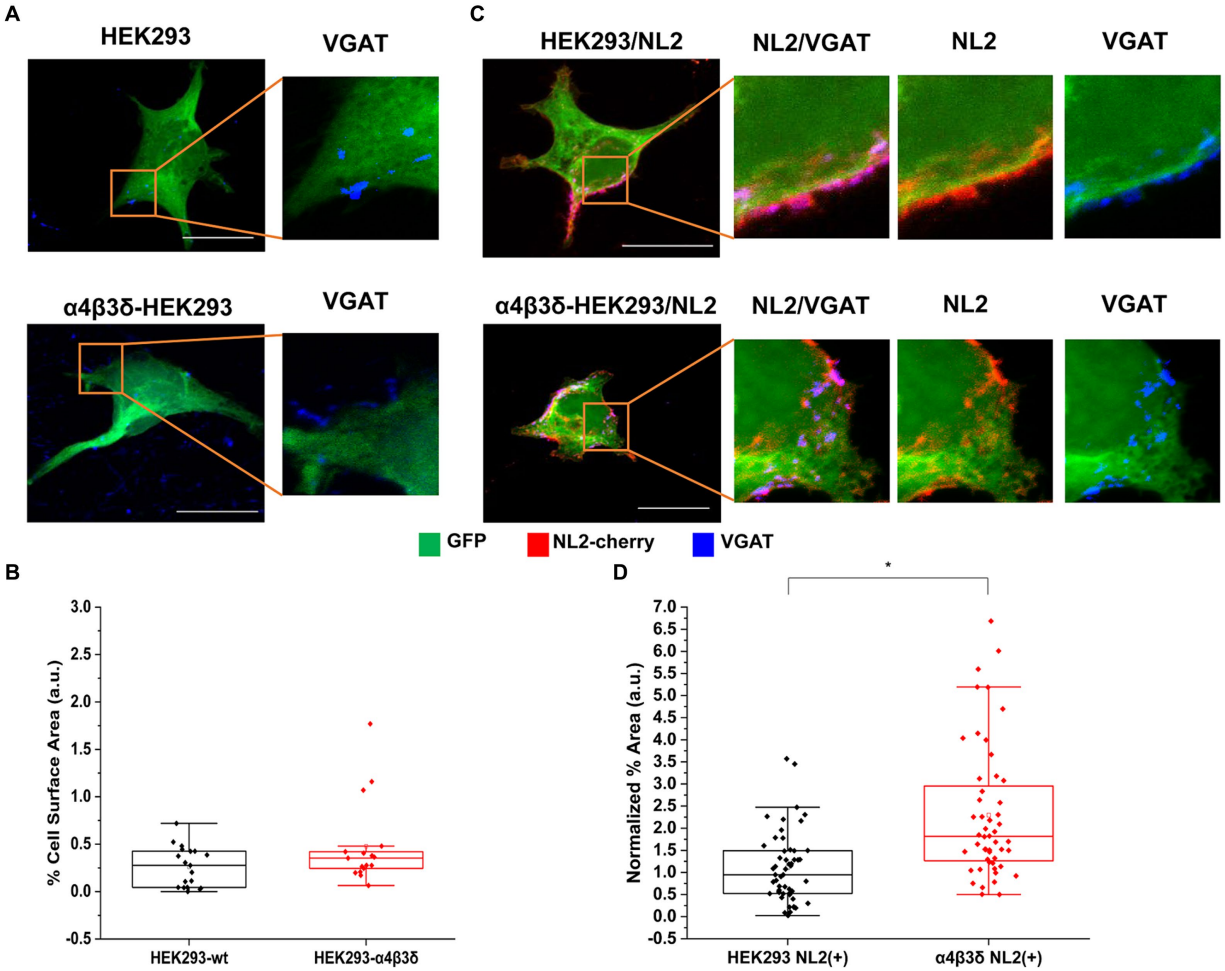
α4β3δ-GABA_A_Rs do not induce synaptic contact formation but potentiate the induction of contacts by NL-2. Synaptic contact formation in co-culture of embryonic medium spiny neurons and **(A)** HEK293 (wt; upper panels) or α4β3δ-GABA_A_R-expressing HEK293 cells (lower panels), or **(B)** HEK293/NL2 cells (wt; upper panels) or α4β3δ-GABA_A_R/NL2-expressing cells (lower panels). The HEK293 cell body was visualized by GFP (green), GABAergic terminals were labeled with an anti-VGAT antibody (blue) and NL2 was mcherry-tagged (red). Scale bar = 20 μm. Fluorescent imaging was done using Zeiss 700 confocal microscope at 63 × magnification with image size 1,024 × 1,024. Max intensity projection of the z-stack images was shown. The enlarged images are 10 × zoom in. Quantitative analysis of synapses expressed as % area of co-localized pixels that represent contacts between VGAT terminals and in **(C)** HEK293 or α4β3δ-GABA_A_R-expressing cells (*n* = 21 and *n* = 17, respectively; *N* = 2 independent experiments, *p* = 0.2), or **(D)** HEK293/NL2 cells or α2β2γ2-GABA_A_R/NL2 expressing cells (*n* = 53 and *n* = 52 cells, respectively; *N* = 3 independent experiments, *p* < 0.00001 (8.4 × 10^−7^)). The box and whisker plot show the mean (square dot with no fill), median (horizontal line), and standard deviation (whiskers). Shapiro–Wilk normality test was used to test the normal distribution of the data and Mann–Whitney test was used to analyze statistical significance of the difference. (*p < 0.05).

The α4β3δ-GABA_A_Rs are generally localized outside of GABAergic synapses and they mediate tonic inhibition (Farrant and Nusser, 2005). These receptors were also expressed in HEK293 cells to create a stable cell line which was characterized using immunolabeling and voltage-clamp electrophysiology. The ubiquitous expression of all three subunits at the cell surface was confirmed by confocal imaging (Supplementary Figures S2A,B). Pharmacological responses of these receptors to GABA and the modulator DS2 (αβδ specific) in whole-cell recordings indicated that the receptors expressing the δ subunit were functional (Supplementary Figures S2C,D). To test whether these receptors can induce the adhesion of GABAergic terminals, the HEK293 cell line or control HEK293 cells were transiently transfected with GFP and co-cultured with the medium spiny neurons (Figure 2A). Quantification of the % area of co-localized pixels which represents contacts between VGAT terminals and HEK293 cells demonstrated no significant difference between the control and α4β3δ-GABA_A_R HEK293 cells (median = 0.28%; IQR = 0.04–0.42%; *n* = 19 cells vs. median = 0.35%; IQR = 0.23–0.45%; *n* = 17 cells, respectively; from *N* = 2 independent experiments, *p* = 0.2, Figure 2B). These results indicate that α4β3δ-GABA_A_Rs do not promote the formation of inhibitory synapses in these cultures, indicating that this activity is a characteristic of the synaptic GABA_A_R subtypes (Brown et al., 2016). However, when α4β3δ-GABA_A_R-HEK293 cells or control HEK293 cells were transiently transfected with GFP and cherry-tagged NL2 cDNAs and co-cultured with the medium spiny neurons (Figure 2C), we detected a significant increase in contacts induced by NL2 in the presence of α4β3δ-GABA_A_Rs (median = 1.81%; IQR = 1.25 - 3.02%; *n* = 52 cells versus the no α4β3δ expressing control median = 0.95%; IQR = 0.52 - 1.49%; *n* = 53 cells, respectively; from N = 3 independent experiments; *p* < 0.00001 (*p* = 8.4 × 10^−7^); Figure 2D). The value of the % area of co-localized pixels for each cell was normalized to the expression of NL2 as described above. These results indicate that extrasynaptic GABA_A_Rs can facilitate the NL2-dependent induction of synapses although their effect is significantly weaker than the effect observed for synaptic GABA_A_Rs.

To test whether the observed potentiation of NL2 effects by GABA_A_Rs may be a consequence of increased expression of NL2, we have transfected the HA-tagged NL2 cDNA into the control, α4β3δ-GABA_A_R- or α2β2γ2-GABA_A_R-expressing HEK293 cells and carried out ELISA using an HA tag-specific antibody (Supplementary Figure S3). However, the surface or total expression of NL2 showed no difference between these conditions, indicating that other molecular mechanisms may mediate the observed cooperative interaction between NL2 and GABA_A_Rs.

**Figure 3:**
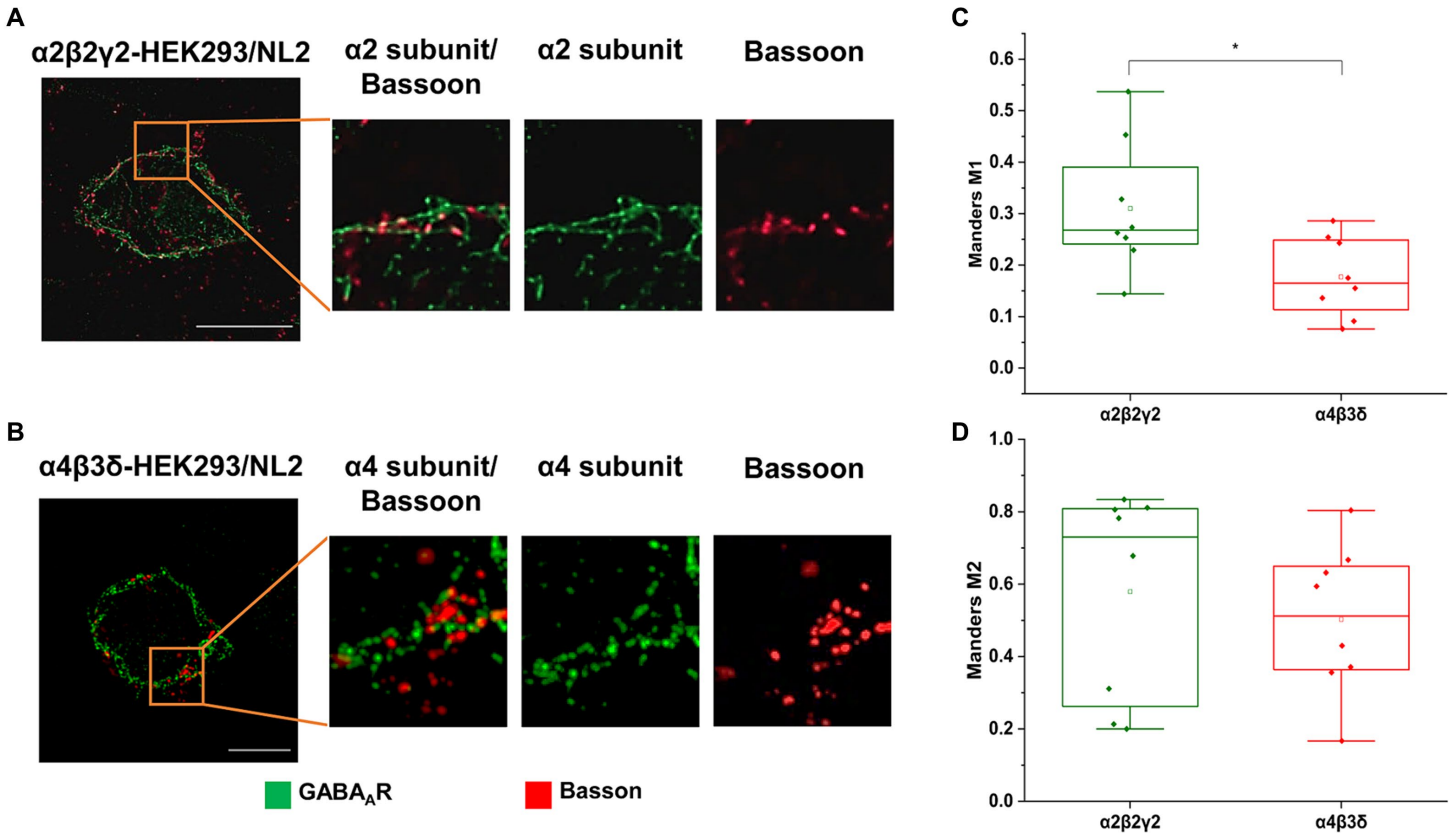
Co-localization of the presynaptic marker Bassoon and GABA_A_ receptors on the surface of **(A)** α_2_β_2_γ_2_-GABA_A_R, or **(B)** α_4_β_3_δ-GABA_A_R-expressing HEK293 cells in co-culture with the embryonic medium spiny neurons. GABA_A_R α_2_ or α_4_ subunits (green) and Basson (red) were visualized using specific antibodies. Images were acquired using Zeiss ELYRA PS.1 SIM at 63 x magnification. Scale bar = 10 μm. **(C,D)** Quantitative analysis of colocalization between presynaptic marker Bassoon and GABA_A_R α2/4 subunits using Manders’ coefficient M1, which indicates the proportion of Bassoon signals that overlap with GABA_A_ receptor α2/4 subunits **(C)**, and Manders’ coefficient M2, which indicates the proportion of α2/4 subunit signals that overlap with Bassoon signals **(D)**. The M1 coefficient is significantly higher in α2β2γ2-GABA_A_R/NL2 expressing cell than in α4β3δ-GABA_A_R/NL2 expressing cells (*t*-test, *p* = 0.04). The M2 coefficient is higher in α2β2γ2-GABA_A_R cells than in α4β3δ-GABA_A_R cells, but this difference is not statistically significant. Data from n = 8 cells from *N* = 2 independent experiments.

The GABA_A_R- and NL2- induced synaptic contacts were further characterized using immunolabeling and super-resolution imaging to measure the degree of co-localization between the α2β2γ2-GABA_A_Rs or α4β3δ-GABA_A_Rs and the presynaptic active vesicular release zone protein Bassoon. In these experiments, the α2β2γ2-GABA_A_R/NL2- or α4β3δ-GABA_A_R/NL2-expressing HEK293 cells in co-culture with the medium spiny neurons were immunolabeled with an α2- or α4-subunit-specific antibody, respectively, in combination with a Bassoon-specific antibody. While both GABA_A_R subtypes showed predominantly punctate distribution at the cell surface, their colocalization with Basson-positive terminals appeared greater for the α2β2γ2-GABA_A_R than α4β3δ-GABA_A_R, which was in agreement with our confocal imaging data (Figures 3A,B). This was confirmed by a significantly higher M1 coefficient value obtained for the Bassoon/α2β2γ2-GABA_A_R co-localization in synaptic contacts than Bassoon/α4β3δ-GABA_A_Rs co-localization (median = 0.27; IQR = 0.24–0.42; *n* = 8 cells versus median = 0.17; IQR = 0.10–0.25; *n* = 8 cells, respectively; from *N* = 2 independent experiments, *p* = 0.04; Figure 3C). In contrast, the M2 coefficient appeared higher but without reaching significance for the α2β2γ2-GABA_A_R overlapping with Bassoon than the α4β3δ-GABA_A_R (median = 0.73; IQR = 0.24–0.81; *n* = 8 cells vs. median = 0.51; IQR = 0.36–0.66; *n* = 8 cells, respectively; from *N* = 2 independent experiments; Figure 3D). These results indicate that NL2-induced formation of synapses is more likely to occur in the proximity of the α2β2γ2-GABA_A_R than α4β3δ-GABA_A_R.

### 3.2 Structural and functional characterization of synaptic contacts induced by co-expression of GABA_A_Rs and NL2

**Figure 4:**
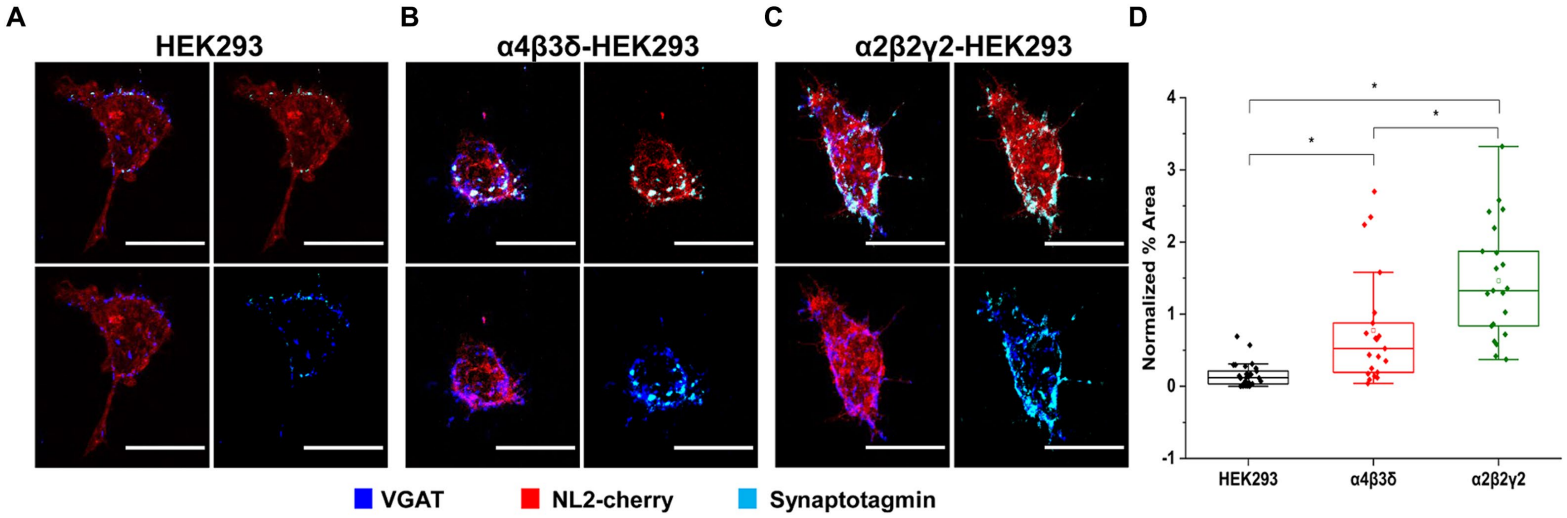
GABA_A_R and NL2 co-expression induces the adhesion of active GABAergic terminals. Presynaptic activity is detected in synapses formed with **(A)** wt/NL2-expressing HEK293 cells, **(B)** α4β3δ-GABA_A_R/NL2-expressing HEK293 cells, or **(C)** α2β2γ2/NL2-GABA_A_R-expressing HEK293 cells in co-culture with embryonic medium spiny neurons. Active presynaptic terminals were visualized with the Cy5-labeled anti-synaptotagmin luminal domain-specific antibody (cyan), while all presynaptic terminals were visualized with an anti-VGAT-specific antibody (blue). NL2 was tagged with mCherry tag (red). Scale bar = 20 μm. Fluorescent imaging was done using Zeiss 700 confocal microscope at 63 × magnification with image size 512 × 512. Max intensity projection of the z-stack images was shown. The enlarged images are 10 × zoom in. **(D)** Quantitative analysis of active synaptic contacts in which the % area was normalized to the expression level of NL2. The box and whisker plot shows the mean (square dot with no fill), median (horizontal line), and standard deviation of the mean (whiskers). Data from HEK293 cells (*n* = 30, from *N* = 3 independent experiments), α4β3δ-GABA_A_R-expressing HEK293 cells (*n* = 22, from *N* = 2 independent experiments) and α2β2γ2-GABA_A_R-expressing HEK293 cells (*n* = 22, from *N* = 2 independent experiments). Significant difference was detected between HEK293 cells and α4β3δ-GABA_A_R-expressing HEK293 cells (*p* = 0.0006), HEK293 cells and α2β2γ2-GABA_A_R-expressing HEK293 cells (6.4 × 10^−10^) and α4β3δ-GABA_A_R-expressing HEK293 cells and α2β2γ2-GABA_A_R-expressing HEK293 cells (*p* = 0.04). Shapiro–Wilk normality test was used to test the normal distribution of the data and Kruskal Wallis ANOVA followed by Dunn’s test was used to analyze the statistical significance (*p < 0.05).

Functional characterization of synaptic contacts formed with the control/NL2, α2β2γ2-GABA_A_R/NL2- or α4β3δ-GABA_A_R/NL2-expressing HEK293 cells was first carried out by assessing the activity of their presynaptic components, GABAergic terminals, in synaptotagmin-antibody uptake assay (Fernández-Alfonso, Kwan and Ryan, 2006). In this assay, active presynaptic terminals were fluorescently labeled with a Cy5-tagged anti-synaptotagmin 1 vesicle-luminal domain-specific antibody (1,50, see Methods). The cells were fixed, permeabilized and immunolabeled with the VGAT-specific antibody, allowing us to visualize both active and inactive terminals forming contacts with HEK293 cells and quantify their ratio (Figures 4A–C). Quantification of the % area of co-localized pixels that represent contacts between synaptotagmin-positive and VGAT-positive terminals and the control HEK293 cells, confirmed that NL2 can induce the adhesion of active terminals in the absence of GABA_A_Rs (median = 0.12%; IQR = 0.03 - 0.22%; *n* = 30 cells; from *N* = 3 independent experiments; Figures 4A,D). However, in the presence of α4β3δ-GABA_A_Rs, NL2 had a significantly greater effect (median = 0.52%; IQR = 0.18 - 0.95%; *n* = 21 cells; from *N* = 2 independent experiments, *p* = 0.0006; Figures 4B,D). Moreover, in the presence of α2β2γ2-GABA_A_Rs, NL2 was even more effective and the number of active synapses was significantly larger [*p* < 0.00001 (*p* = 6.4 × 10^−10^)] than the number obtained in the absence of GABA_A_Rs or presence of α4β3δ-GABA_A_Rs (median = 1.32%; IQR = 0.78 - 2.03%; *n* = 21 cells; from *N* = 2 independent experiments, *p* = 0.04, Figures 4C,D). The percentage of synapses incorporating active terminals in these experiments was ~10% (control/NL2), 24% (α4β3δ-GABA_A_R/NL2), and 30% (α2β2γ2-GABA_A_R/NL2).

**Figure 5:**
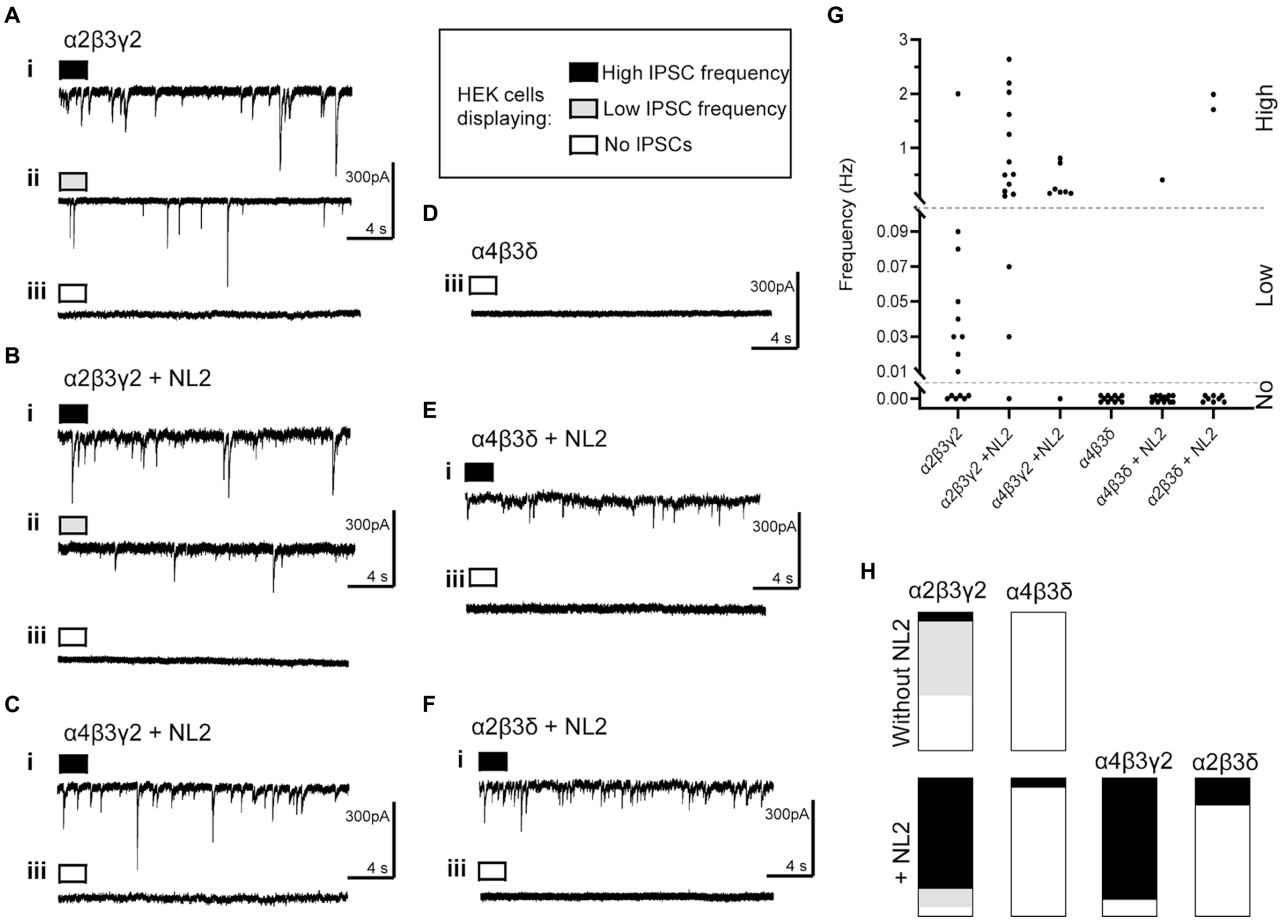
Whole cell recordings of IPSCs in co-culture of HEK293 cells and embryonic medium spiny neurons. Representative examples of voltage-clamp recordings of IPSCs in HEK cells expressing **(A)** α2β3γ2-GABA_A_Rs without NL2, **(B)** α2β3γ2 with NL2, **(C)** α4β3γ2 with NL2, **(D)** α4β3δ without NL2, **(E)** α4β3δ with NL2, and **(F)** α2β3δ with NL2. Examples of three levels of IPSC frequency are shown as (i) high, >0.1 Hz, black; (ii) low, <0.1 Hz, gray; or **(iii)** no IPSCs, white. **(G)** Scatter-plot showing individual IPSC frequencies in each group. **(H)** Parts-of-whole histograms show the prevalence of observing a high, low or zero IPSC frequency, with the following number of cells appearing in each plot (high/low/none-total): α2β3γ2-NL2: 1/8/6–15, α2β3γ2 + NL2: 12/2/1–15, α4β3γ2 + NL2: 7/0/1–8, α4β3δ-NL2: 0/0/11–11, α4β3δ + NL2: 1/0/14–15, α2β3δ + NL2: 2/0/8–10.

Electrophysiological analysis of the whole-cell recordings of inhibitory postsynaptic currents (IPSCs) in GABA_A_R- or GABA_A_R/NL2 expressing HEK293 cells revealed that GABA-mediated synaptic transmission could be detected reproducibly only in the presence of the α2β2γ2-GABA_A_Rs (Figure 5). In the absence of NL2, the IPSCs were detected in 60% of the α2β2γ2-GABA_A_R-HEK293 cells (Figures 5A,G,H; 9 out of 15), in which 8 cells were detected with <0.1 Hz IPSC and 1 cell with >0.1 Hz, which supports the results of the structural analysis presented in Figure 1A. When NL2 was co-expressed, IPSCs were detected in 93.3% of α2β2γ2-GABA_A_R/HEK293 cells (14 out of 15), in which 2 cells were detected with <0.1 Hz IPSC and 12 cells with >0.1 Hz (Figures 5B,G,H). In the absence of NL2, no IPSCs were detected in the α4β3δ-GABA_A_R/HEK293 cells (11 cells, Figures 5D,G,H), while in the presence of NL2, IPSCs were detected in only 1 α4β3δ-GABA_A_R-HEK293 cell out of 15 (> 0.1 Hz; Figures 5E,G,H). These data show that expression of NL2 increases the frequency of GABAergic IPSCs in the presence of synaptic α2β2γ2-GABA_A_Rs. However, synaptic contacts induced by NL2 alone or the presence of α4β3δ-GABA_A_Rs (Figures 2–4) fail to differentiate into active synapses and remain functionally silent (~95% of cells; 1/15).

Given a clear difference in the number and activity of NL2-induced synaptic contacts in the presence of α2β2γ2- and α4β3δ-GABA_A_Rs, we were keen to establish which of the GABA_A_R subunits may play a key role in these processes. To test different subunit combinations, we transiently transfected into the β3-expressing HEK293 stable cell line (B2 clone) combinations of α2, α4, γ2 or δ subunits cDNAs to form the following GABA_A_R subtypes: α2β3γ2, α4β3δ, α4β3γ2 or α2β3δ. These cells were also co-transfected with cherry-NL2 cDNA. The cells were co-cultured with medium spiny neurons and synaptic activity in HEK293 cells was examined using whole-cell recordings. Compared to the α2β2γ2-GABA_A_R/NL2-expressing HEK293 cells, a similar level of activity was detected in 7 out of 8 of the α4β3γ2-GABA_A_R/NL2-expressing HEK293 cells with IPSC frequencies >0.1 Hz (Figures 5C,G,H). However, in 8 out of 10 of the α2β3δ-GABA_A_R/NL2-expressing HEK293 cells, no IPSCs were detected, indicating that the majority of synaptic contacts formed in these conditions were also functionally silent (Figures 5F–H).

**Figure 6:**
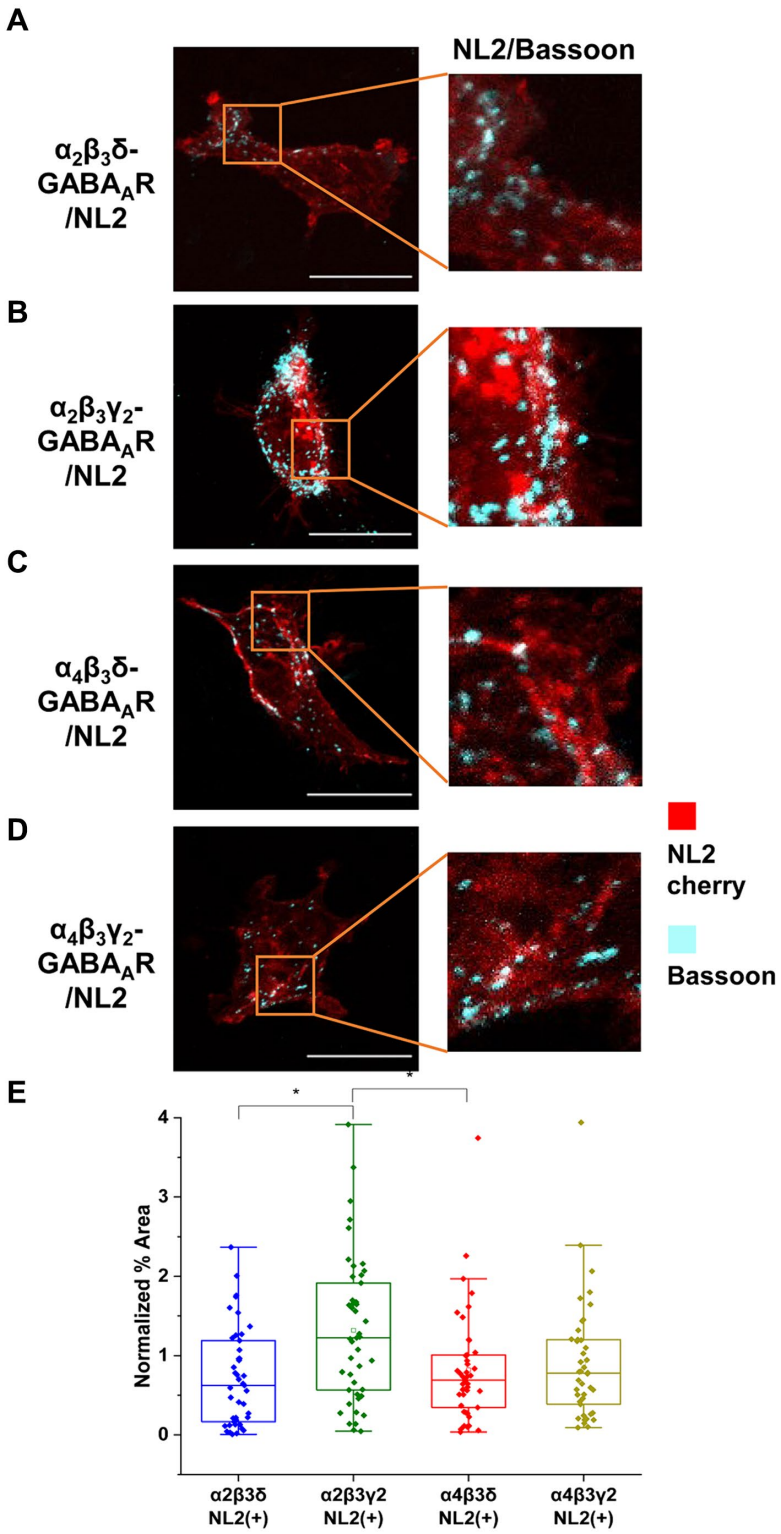
α2β3γ2-GABA_A_R and NL2 co-expression is the most potent combination in inducing synaptic contacts. HEK293 cells expressing **(A)** α2β3δ-, **(B)** α2β3γ2-, **(C)** α4β3δ-, or **(D)** α4β3γ2-GABA_A_R and NL2 were co-cultured with embryonic medium spiny neurons. NL2 was tagged with mCherry (red) while GABAergic terminals were labeled an anti-Bassoon-specific antibody (cyan). Scale bar = 20 μm. Fluorescent imaging was done using 63 × magnification with image size 1,024 × 1,024. Max intensity projection of the z-stack images was shown. The enlarged images are 10 × zoom in. **(E)** Quantitative analysis of synapses expressed as % area of colocalised pixels that represent contacts between Basson-positive terminals and HEK293 expressing α2β3δ-, α2β3γ2-, α4β3δ- or α4β3γ2-GABA_A_ receptors and NL2. The % area was normalized to the expression level of NL2 for each cell. The box and whisker plot shows the mean (square dot with no fill), median (horizontal line), and standard deviation of the mean (whiskers), with filled dots representing individual cells. Data from *N* = 3 independent experiments. Significant difference was detected between α2β3γ2-GABA_A_R- and α2β3δ-GABA_A_R-expressing HEK293 cells (*p* = 0.003) or α4β3δ-GABA_A_R-expressing HEK293 cells (*p* = 0.03). Shapiro–Wilk normality test was used to test the normal distribution of the data and Kruskal Wallis ANOVA followed by Dunn’s test was used to analyze the statistical significance of the difference. (*p < 0.05).

To investigate these differences further, we have also carried out immunolabeling experiments to characterize the degree of innervation of HEK293 cells expressing different subunit combinations (Figures 6A–D). Synaptic contacts were defined based on signal colocalization between the presynaptic Bassoon and postsynaptic NL2 (cyan and red channel in Figures 6A–D) and analyzed using ImageJ as described in the Methods. Quantification of the % area of co-localized pixels that represent contacts between the Bassoon-positive terminals and NL2 demonstrated that the level of presynaptic innervation was significantly higher in the presence of α2β3γ2-GABA_A_Rs (median = 1.22%; IQR = 0.54 – 1.96%; *n* = 45) than α2β3δ- (median = 0.62%; IQR = 0.17 – 1.19%; *n* = 43, *p* = 0.003) or α4β3δ- GABA_A_Rs (median = 0.69%; IQR = 0.34 – 1.01%; *n* = 43, *p* = 0.03) (from *N* = 3 independent experiments; Figure 6E). The lower innervation for α4β3γ2 combination, detected by imaging, was surprising given its ability to mediate IPSCs in the presence of NL2 (Figures 5C,G,H), possibly reflecting the greater sensitivity of electrophysiology for detecting active synapses.

Together, our results point to a key role of the γ2 subunit in facilitating the NL2-induced synapse formation and functional maturation in this co-culture model. Although adhesion of active GABAergic terminals can be induced by NL2 in the presence of other GABA_A_R subunit combinations, the highest degree of innervation and the tight functional coupling between the presynaptic release of GABA and the postsynaptic responses require the cooperation between the γ2 subunit-containing GABA_A_Rs and NL2.

### 3.3 The synergism between GABA_A_Rs and NL2 does not require GABA_A_R activity

To test if the GABA_A_R activation by GABA may be required for the observed effects, the control, α2β2γ2-GABA_A_R or α4β3δ-GABA_A_R-expressing HEK293 cells were transiently transfected with GFP and cherry-NL2 cDNAs and platted together with the medium spiny neurons in the absence or presence of bicuculline, a GABA_A_R competitive antagonist (Supplementary Figure S4). Quantification of the % area of co-localized VGAT and GFP pixels that represent contacts showed potentiation of NL2 effects by α2β2γ2-GABA_A_Rs irrespective of whether the cultures were incubated with DMSO (control HEK293/NL2 cells: median = 1.68%; IQR = 0.54 – 2.57%; *n* = 20; α2β2γ2-GABA_A_R/NL2 cells: median = 4.21%; IQR = 2.00 – 7.11%; *n* = 20; from *N* = 2 independent experiments, *p* = 0.01) or bicuculline (control HEK293/NL2 cells: median = 1.11%; IQR = 0.43 – 2.00%; *n* = 21; α2β2γ2-GABA_A_R/NL2 cells: median = 2.28%; IQR = 1.34 – 4.22%; *n* = 20, respectively; from *N* = 2 independent experiments, *p* = 0.009; Supplementary Figures S4A,C).

Similar results were obtained in co-cultures of control HEK293/NL2 and α4β3δ-GABA_A_R/NL2-expressing HEK293 cells in the absence or presence of bicuculline (Supplementary Figures S4B,D). Quantification of the % area of co-localized pixels of VGAT and GFP that represent contacts between the presynaptic terminals and HEK293 cells showed a significant increase in NL2 induction in the presence of α4β3δ-GABA_A_Rs in both DMSO-treated cultures (control HEK293/NL2 cells: median = 1.05% IQR = 0.52 – 1.72%; *n* = 20; α4β3δ-GABA_A_R/NL2 cells: median = 2.89% IQR = 1.77 – 4.99%; *n* = 20; from *N* = 2 independent experiments, *p* = 0.03) and bicuculline-treated cultures (control HEK293/NL2 cells: median = 1.05% IQR = 0.27 – 1.27%; *n* = 20; from *N* = 2 independent experiments; α4β3δ-GABA_A_R/NL2 cells: median = 3.23% IQR = 1.36 – 4.79%; *n* = 20; from *N* = 2 independent experiments, *p* = 0.03; Supplementary Figure S4D).

### 3.4 GABA_A_Rs and NL2 synergism is mediated by the TM3-4 intracellular loop of the γ2 subunit

**Figure 7:**
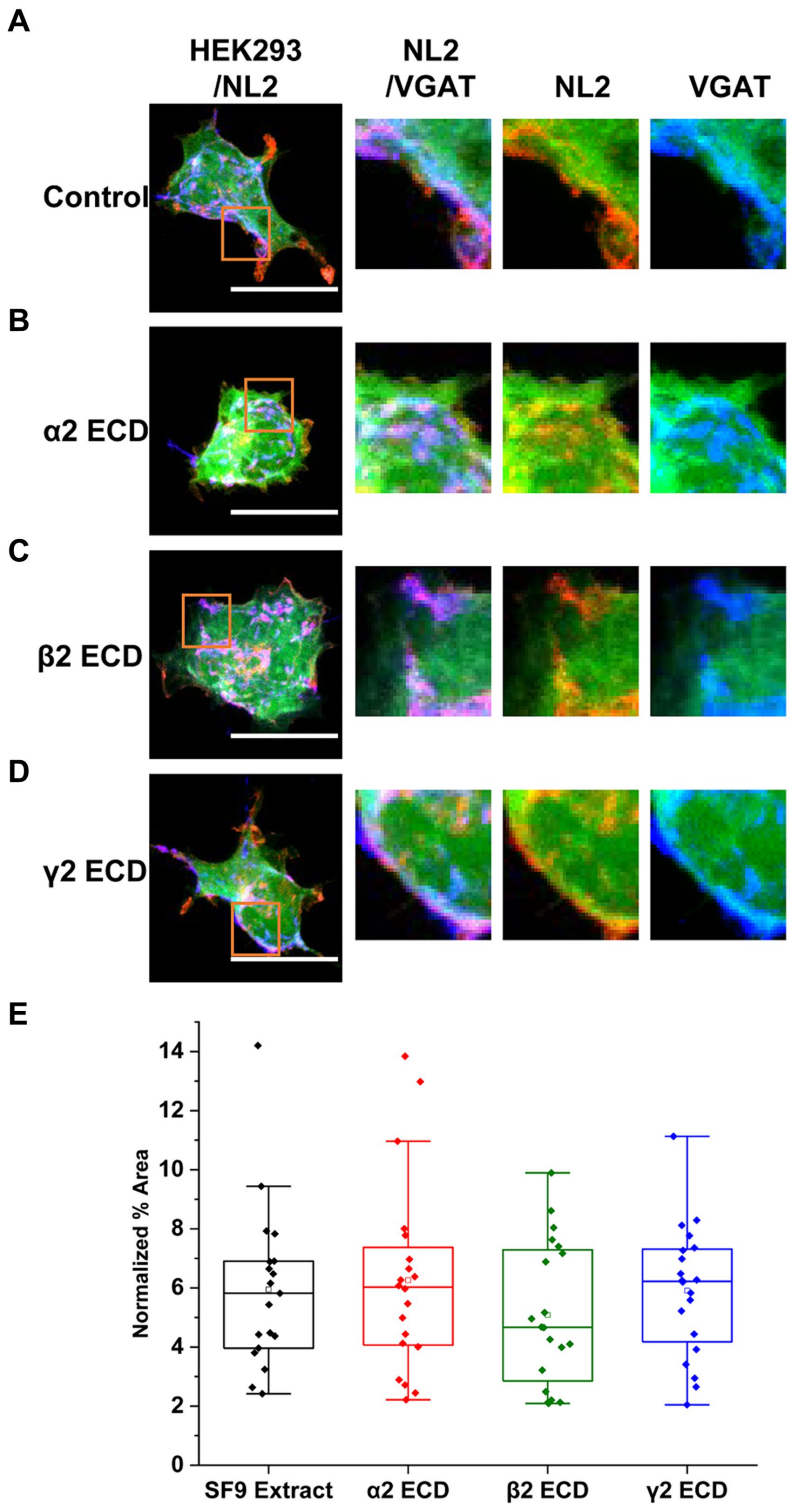
The N-terminal extracellular domains (ECDs) of GABA_A_R subunits do not mediate the induction of synaptic contacts by GABA_A_R and NL2 co-expression. **(A)** Synaptic contact formation in co-culture of α2β2γ2-GABA_A_R/mCherry-NL2/GFP–expressing HEK293 cells and embryonic medium spiny neurons in the presence of **(A)** SF9 cell extracts, **(B)** α2 subunit ECD, **(C)** β2 subunit ECD or **(D)** γ2 subunit ECD. HEK293 cell body was visualized with GFP (green), NL2 was labeled with mCherry (red), and the presynaptic terminals were visualized with an anti-VGAT-specific antibody (blue). Scale bar = 20 μm. Fluorescent imaging was done using 63 × magnification with image size 512 × 512. Max intensity projection of the z-stack images was shown. The enlarged images are 10 × zoom in. **(E)** Quantitative analysis of the % area of synaptic contacts formed with α2β2γ2-GABA_A_ receptor/NL2-expressing HEK293 cells. The % area values were normalized to the expression of NL2 for each cell. The box and whisker plot shows the mean (square dot with no fill), median (horizontal line), and standard deviation of the mean (whiskers). Data from *n* = 19 α2β2γ2-GABA_A_/NL2-expressing HEK293 cells treated with SF9, *n* = 20 treated with α2 subunit ECD, *n* = 20 treated with β2 subunit ECD, and *n* = 20 treated with γ2 subunit ECD; from *N* = 2 independent experiments. Shapiro–Wilk normality test was used to test the normal distribution of the data and Kruskal Wallis ANOVA followed by Dunn’s test was used to analyze the statistical significance of the difference.

To investigate the mechanisms underlying the observed synergistic effects of α2β2γ2-GABA_A_Rs and NL2 in synapse formation, we first assessed whether the extracellular N-terminal domains (ECDs) of GABA_A_R α2, β2 or γ2 subunits may be involved given that they were previously shown to contribute to the GABAergic synapse formation in the absence of NL2 (Figure 7; Brown et al., 2016).

To test this, 4 μg of either the α2 (Figure 7B), β2 (Figure 7C), or γ2 ECD (Figure 7D; 0.29–0.32 μM), purified from SF9 cells (Brown et al., 2016) were applied to the co-culture of α2β2γ2-GABA_A_R/GFP/NL2-expressing HEK293 cells and medium spiny neurons. An equivalent amount of untransfected SF9 cell extract (4 μg), following the same purification procedure as the extracts expressing ECDs, was used as a control (Figure 7A). Quantification of the % area of co-localized pixels of VGAT and GFP that represent synaptic contacts showed no significant change with the application of ECDs (Figure 7E). With SF9 extract control, the median was 5.82% (IQR = 3.96 - 6.90%, n = 19 cells from N = 2 independent experiments). Application of β2 ECD slightly decreased the synapse formation albeit not significantly (median = 4.67%; IQR = 2.67 – 7.35%; *n* = 20 cells; from *N* = 2 independent experiments). No change was observed with the application of α2 (median = 6.03%; IQR = 4.04 – 7.58%; *n* = 20 cells; from *N* = 2 independent experiments) or γ2 (median = 6.22%; IQR = 4.05 – 7.33%; *n* = 20 cells; from *N* = 2 independent experiments) ECDs.

**Figure 8:**
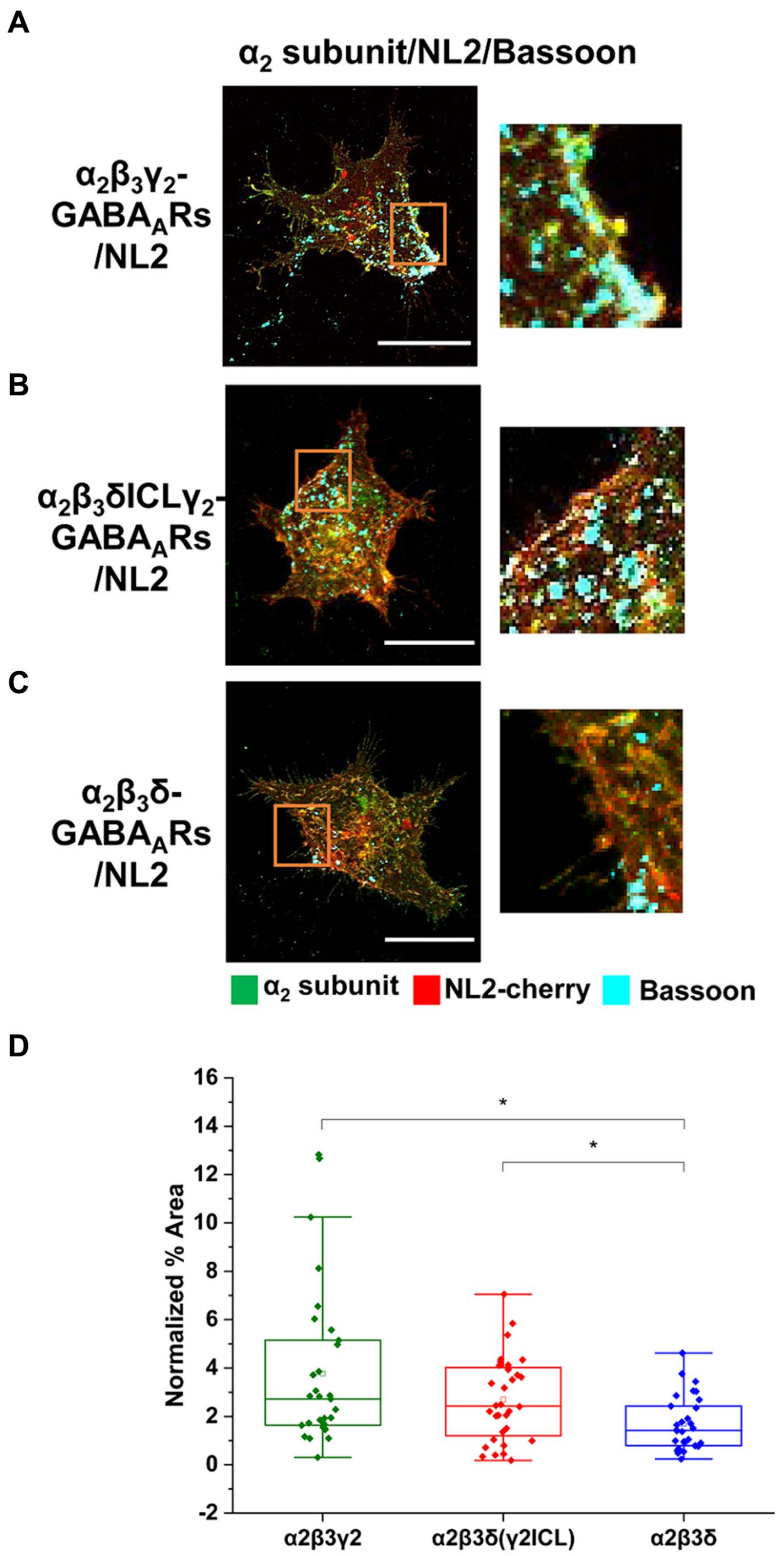
Cooperative interaction between GABA_A_Rs and NL2 is mediated by the γ2 subunit intracellular loop. **(A)** Synaptic contact formation in co-culture of α2β3γ2-GABA_A_R/NL2-, **(B)** α2β3δγ2ICL-GABA_A_R/NL2- or **(C)** α2β3δ-GABA_A_R/NL2-expressing HEK293 cells and embryonic medium spiny neurons. NL2 was tagged with mCherry (red), synaptic terminals were labeled with an anti-Bassoon antibody (cyan), and GABA_A_Rs were visualized with an anti-α2 subunit specific antibody (green). Scale bar = 20 μm. Fluorescent imaging was done using Zeiss 700 confocal microscope at 63 × magnification with image size 512 × 512. Max intensity projection of the z-stack images was shown. The enlarged images are 10 × zoom in. **(D)** Quantitative analysis of the % area of synaptic contacts which was normalized to the expression of NL2 for each cell. The box and whisker plot shows the mean (square dot with no fill), median (horizontal line), and standard deviation of the mean (whiskers). Data from *n* = 31 α2β3γ2-GABA_A_R/NL2-, *n* = 32 α2β3δγ2ICL-GABA_A_R/NL2-, and *n* = 30 α2β3δ-GABA_A_R/NL2-expressing HEK293 cells, from *N* = 2 independent experiments. Significant difference was detected between α2β3γ2-GABA_A_R/NL2- and α2β3δ-GABA_A_R/NL2-expressing HEK293 cells (*p* = 0.003) or α2β3δγ2ICL- and α2β3δ-GABA_A_R/NL2-expressing HEK293 cells (*p* = 0.047). Shapiro–Wilk normality test was used to test the normal distribution of the data and Kruskal Wallis ANOVA followed by Dunn’s test was used to analyze the statistical significance of the difference. (*p < 0.05).

These results indicate that cooperation between α2β2γ2-GABA_A_Rs and NL2 may be mediated by the subunit intracellular domains. To test this hypothesis, we took advantage of previously characterized δ-γ2 subunits chimera (Hannan et al., 2020), in which the large intracellular loop (ICL) (TM 3–4) of the δ subunit was replaced with the equivalent domain of the γ2 subunit (δγ2ICL). HEK293 cells stably expressing β3 subunits were transiently transfected with the cherry-NL2 and α2 + γ2 (Figure 8A), or α2 + δγ2ICL (Figure 8B), or α2 + δ (Figure 8C) cDNAs and cultured with medium spiny neurons for 24 h. Quantification of the % area of co-localized pixels that represent contacts between presynaptic Bassoon and NL2 demonstrated no significant difference in NL2-dependent induction of synaptic contacts between the α2β3γ2/NL2 and α2β3δγ2ICL/NL2 (median = 2.72%; IQR = 1.63 – 5.15%; *n* = 31 cells, versus median = 2.42%; IQR = 1.12 – 4.06%; *n* = 32 cells; *N* = 2 independent experiments). In both conditions, the NL2 effects were significantly greater than in the presence of α2β3δ-GABA_A_Rs (median = 1.42%; IQR = 0.78 – 2.49%; *n* = 30 cells, respectively, *p* = 0.003 for α2β3γ2/NL2, *p* = 0.047 for α2β3δγ2ICL/NL2; *N* = 2 independent experiments; Figure 8D). Thus, the TM3-4 intracellular loop of the γ2 subunit mediates the cooperativity between GABA_A_Rs and NL2. Whether the large intracellular loop of the γ2 subunit mediated a direct interaction between the GABA_A_Rs and NL2 remained unclear.

**Figure 9:**
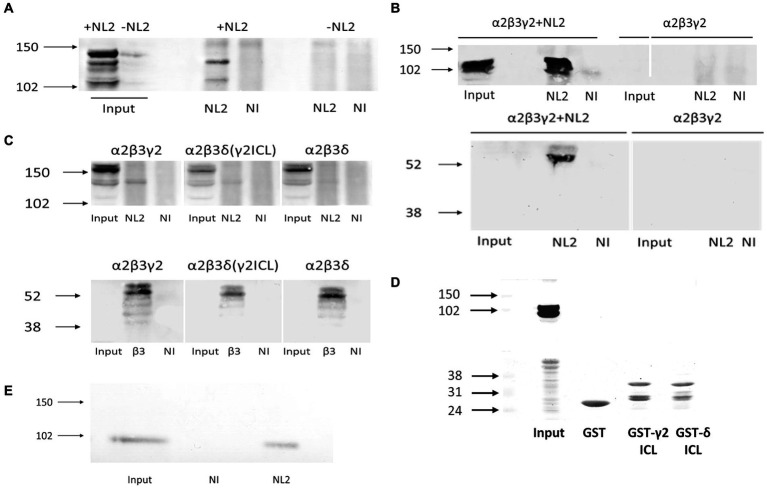
GABA_A_R and NL2 interaction is mediated by the γ2 subunit intracellular loop. **(A)** Myc-tagged GABA_A_Rs were immunoprecipitated using a myc-specific antibody, followed by detection of NL2 by immunoblotting using an anti-NL2-specific antibody. **(B)** HA-tagged NL2 was immunoprecipitated with a HA-specific antibody and subsequently detected by immunoblotting with the same antibody (upper panel), while the presence of GABA_A_Rs in precipitates was detected by immunoblotting with an anti-β3 specific antibody (lower panel). **(C)** NL2-GABA_A_ receptor interaction in the presence of the γ2 subunit intracellular TM 3–4 loop domain detected by co-immunoprecipitation. The β3 subunit was myc-tagged. The immunoprecipitation was carried out with an anti-myc-specific antibody. NL2 was detected in precipitates using an anti-NL2-specific antibody (upper panel), while GABA_A_ receptors were detected using an anti-β3 subunit-specific antibody (lower panel). **(D)** NL2 does not bind to the purified GST-γ2 ICL or GST-δ ICL *in vitro* (upper panel). Purified GST fusion proteins were detected by Ponceau S (lower panel). **(E)** NL2 interacts with GABA_A_Rs in the adult male rat cortex. Co-immunoprecipitation was carried out with an anti-α1 subunit C-terminal-specific antibody, followed by detection of NL2 by immunoblotting using an anti-NL2-specific antibody. In all immunoblotting experiments, the binding of primary antibodies was visualized using alkaline phosphatase-conjugated secondary antibody and NBT/BCIP color substrate reaction. Blots are representative of *N* = 2 independent experiments for each experimental paradigm.

To address this question, we have carried out co-immunoprecipitation experiments using the lysates of HEK293 cells expressing Myc-tagged α2β3γ2-GABA_A_Rs and HA-tagged NL2. Using either the Myc-antibody, followed by immunoblotting with the NL2-specific antibody (Figure 9A), or, conversely, HA-tag antibody, followed by immunoblotting with β3 subunit-specific antibody (Figure 9B), we confirmed that GABA_A_Rs and NL2 can be co-immunoprecipitated and thus can interact with each other. Furthermore, to assess if the ICL of γ2 subunit mediates this interaction, α2β3γ2-, or α2β3δγ2ICL- or α2β3δ-GABA_A_Rs were co-expressed with HA-tagged NL2 in HEK293 cells, and subjected to co-immunoprecipitation with the Myc-antibody followed by immunoblotting with the NL2-antibody or β3-subunit antibody. In α2β3γ2-GABA_A_R precipitates, a clear band corresponding to the molecular weight for NL2 was detected while no such band was detected in α2β3δ-GABA_A_R precipitates (Figure 9C, upper panel). In the α2β3δγ2ICL-GABA_A_R precipitates, a weaker band corresponding to NL2 was also detected (Figure 9C, upper panel). Immunoblotting with the β3 subunit antibody showed that the amount of precipitated GABA_A_Rs was comparable in three different conditions (Figure 9C, lower panel). To test if NL2 can bind directly to the large intracellular domain of the γ2 subunit, GST-tagged ICL was expressed and purified from *E. coli* and incubated with the lysates of HEK293 cells transfected with HA-NL2. However, the binding between NL2 and the TM 3–4 ICL of the γ2 subunit (Figure 9D) could not be detected, indicating that GABA_A_Rs and NL2 interaction may be indirect and likely mediated by another protein, the nature of which remains to be established. Nevertheless, GABA_A_Rs and NL2 can interact *in vivo* as demonstrated by their co-immunoprecipitation from rat brain lysates prepared under non-denaturing conditions (Figure 9E).

## 4 Discussion

GABA_A_Rs and NL2 are co-expressed in many GABAergic inhibitory synapses in the mammalian brain with both proteins being implicated in synaptic initiation and functional maturation (Ali et al., 2020). It is now evident that together with many other proteins, GABA_A_Rs and NL2 undergo complex interactions to facilitate synaptic contact formation but how these interactions are coordinated in time and space to lead to the establishment of fully functional inhibitory synapses remains to be described in detail. This is important because genetic mutations and alterations in NL2 and GABA_A_Rs expression and function found in patients have been directly associated with their neurological and psychiatric symptoms, often showing a degree of overlap (Ali et al., 2020; Thompson, 2024). In many such cases, deficits in inhibitory synaptic connections have been implicated as the leading cause of the symptoms that patients experience. However, the intricate details of molecular interactions with a clear functional read-out are difficult to study in complex *in vivo* or *in vitro* systems in which these and many other proteins coexist. The current study therefore aimed to investigate whether and how the interaction between GABA_A_Rs and NL2 may lead to the establishment of functional synapses in the absence of other synaptic proteins using a reduced *in vitro* co-culture system. Although far from the situation *in vivo* and subject to the limitations of any study in a reduced system, this approach has revealed a synergism between GABA_A_Rs and NL2 in inducing synaptic formations for which the presence of the γ2 subunit and specifically its TM3-4 intracellular domain-mediated interaction between these proteins are required.

We also know that the phosphorylation status of NL2 is important for its surface stability and for regulating synaptic GABA_A_R numbers at synapses (Halff et al., 2022). Furthermore, we and others have previously shown that the number of synaptic contacts could be enhanced significantly by co-expression of NL2 and GABA_A_Rs in heterologous co-culture models (Fuchs et al., 2013) and cultured neurons (Fu and Vicini, 2009). Moreover, in functional experiments, synaptic responses including spontaneous IPSC and miniature IPSC amplitudes detected in the presence of NL2 (and GABA_A_Rs) indicated that each nerve terminal elicits a more efficacious postsynaptic response and that each axon forms more functional synapses. Our current study largely confirms these findings but also draws an important distinction between the synaptic and extrasynaptic subtypes of GABA_A_Rs in their ability to synergize with NL2. The synaptic GABA_A_Rs, those more closely associated with NL2 *in vivo*, show a significantly stronger synergism with NL2 in synaptic initiation and pre-and post-synaptic coupling leading to the formation of fully functional synapses. The extrasynaptic δ-GABA_A_Rs, although able to potentiate the synaptogenic effects of NL2 to some extent, do not generate the same postsynaptic responses as their γ2 counterparts, probably due to their largely perisynaptic localization rather than their intrinsic channel properties given that they have a higher affinity for GABA and slower desensitization rate than synaptic GABA_A_Rs (Farrant and Nusser, 2005; Mortensen et al., 2011). This infers that synaptic GABA_A_Rs may have a stronger physical association with NL2 than the extrasynaptic receptors which is indeed supported by our biochemical experiments showing that NL2 could be detected only in precipitated protein complexes of synaptic GABA_A_Rs.

Our current study also confirms the previously reported synaptogenic activity of synaptic GABA_A_Rs in the absence of NL2 (Fuchs et al., 2013; Brown et al., 2014, 2016). These *in vitro* findings are supported by the *in vivo* evidence from GABA_A_R α1, α3 or γ2 subunit knock-out mice demonstrating that the lack of these subunits in certain brain regions leads to prominent structural changes in specific types of inhibitory synapses (Schweizer et al., 2003; Li et al., 2005; Fritschy and Panzanelli, 2006; Studer et al., 2006). Moreover, genetic deletion of GABA_A_Rs using CRISPR-Cas9 technology in a single hippocampal neuron was shown to cause a substantial reduction in GABAergic synapses received by this cell (Duan et al., 2019), further supporting the critical role of GABA_A_Rs in inhibitory synapse development.

NL2 has been viewed as a chief synaptic adhesion mediator based on its well-characterized interactions with the presynaptic proteins Neurexins (Sudhof, 2008), although many other adhesion proteins have also been shown to facilitate the initiation of synaptic contacts (Connor and Siddiqui, 2023). However, the presence of GABA_A_Rs is the key requirement for these contacts to develop into functional synapses. Moreover, in the presence of synaptic GABA_A_Rs and NL2 at the postsynaptic membrane, significantly more presynaptic GABAergic terminals show the activity-dependent uptake of synaptotagmin luminal domain-specific antibodies indicating that their ability to release GABA is enhanced in comparison with the conditions where only NL2 or GABA_A_Rs are expressed. This suggests that GABA_A_Rs also influence presynaptic maturation regulated by NL2 either directly, by interacting with the presynaptic proteins such as Neurexins (Zhang et al., 2010) and/or other proteins involved in this process (Brown et al., 2016), or they act indirectly, by facilitating the NL2 interactions with its presynaptic partners. However, direct interactions of GABA_A_Rs with presynaptic proteins in this context are less likely to contribute because introducing the purified N-terminal ECDs of individual subunits as blocking reagents into our co-cultures did not affect the synergism between GABA_A_Rs and NL2. Nevertheless, it remains possible that GABA_A_Rs may still engage directly via interactions that require the fully assembled heteropentameric N-terminals ECDs rather than ECDs of individual subunits used in our experiments or they may act via their C-terminal ECDs. The indirect facilitation of presynaptic maturation by GABA_A_Rs is supported by our findings as well as by previous studies. Our results indicate that the intracellular TM3-4 loop of the γ2 subunits is required for the cooperativity between GABA_A_Rs and NL2 in synaptic formation but also for the association between GABA_A_Rs and NL2. However, the TM 3–4 ICL may not be sufficient for binding to occur, given that no binding to NL2 was detected when the purified GST-fusion of the γ2 TM3-4 intracellular loop was tested in binding assays *in vitro*. It is therefore likely that this association is mediated by another protein that can bind directly to both the γ2 TM3-4 intracellular loop and NL2. While there may be several proteins involved, one of the two main candidates is the tetraspanin LHFPL4, also known as GARLH4, which interacts with the γ2 subunit to link GABA_A_Rs and NL2 (Davenport et al., 2017; Yamasaki et al., 2017; Han et al., 2021). However, the attempts to detect this protein in our HEK293 cell lines using immunoblotting with specific antibodies were unsuccessful (data not shown), which leads us to conclude that this protein is unlikely to play a role in the synergism between GABA_A_Rs and NL2 observed in this study. The other main candidate for this role is the postsynaptic scaffold protein gephyrin which was shown previously to directly interact with the TM3-4 intracellular loops of multiple GABA_A_Rs subunits, including the γ2 subunit (Tretter et al., 2008; Maric et al., 2011; Mukherjee et al., 2011; Tretter et al., 2011; Kowalczyk et al., 2013; Maric et al., 2014), but also NL2 (Antonelli et al., 2014; Nguyen et al., 2016). Gephyrin is expressed in our HEK293 cell lines in abundance (data not shown) which suggests that the observed interaction between NL2 and GABA_A_Rs may be at least in part mediated by this protein.

It is also likely that multiple interactions between gephyrin and α (1, 2, 3 or 5), β (2 or 3) and γ2 subunits incorporated into the synaptic subtypes of GABA_A_Rs occur at the same time and cumulatively contribute to a strong and stable binding of the receptor to gephyrin and NL2, which may be required for the initiation of synapses. In contrast, the extrasynaptic GABA_A_R subtypes may still engage in interactions with gephyrin and indirectly with NL2 via their β subunits given that α4, α6, or δ do not bind gephyrin, but this interaction is likely to be weaker and transient and therefore insufficient to stabilize the complex between GABA_A_Rs and NL2 to the extent required for the formation of new functional synapses. This could potentially explain the findings that the initiation of contacts is still increased in the presence of extrasynaptic GABA_A_R and NL2, but these contacts do not develop into functional synapses. The perisynaptic localization of these receptors shown in our study and previously (Wei et al., 2003; Farrant and Nusser, 2005) is in agreement with this hypothesis. Moreover, the transient nature of these interactions and the ability of extrasynaptic GABA_A_Rs to migrate laterally into and out of synapses even when synapses are established and fully functional has been demonstrated in a study which also showed that the TM3-4 loop of γ2 subunit plays a key role in regulating the degree of lateral migration of synaptic GABA_A_Rs (Hannan et al., 2020). Finally, the apparent correlation between the degree of synergism between GABA_A_Rs and NL2 and the establishment of functional synapses led us to assess whether GABA_A_R channel activity may contribute to these developmental processes as described previously (Chattopadhyaya et al., 2007; Arama et al., 2015; Oh et al., 2016). In the presence of bicuculline, the synergism between synaptic or extrasynaptic GABA_A_R and NL2 in synaptic contact formation was unaffected which further supports our hypothesis that GABA_A_Rs not only mediate synaptic transmission in the brain but also participate together with NL2 in the initiation and maturation of synaptic contacts as structural proteins.

## Data availability statement

The raw data supporting the conclusions of this article will be made available by the authors, without undue reservation.

## Ethics statement

The animal study was approved by Ethics Committee University College London. The study was conducted in accordance with the local legislation and institutional requirements.

## Author contributions

YS: Writing – review & editing, Methodology, Investigation, Formal analysis, Data curation, Conceptualization. MM: Writing – review & editing, Methodology, Investigation, Formal analysis, Data curation. BY: Writing – review & editing, Methodology, Investigation, Formal analysis. MN: Writing – review & editing, Methodology. TS: Funding acquisition, Writing – review & editing, Supervision, Conceptualization. JJ: Writing – review & editing, Writing – original draft, Supervision, Project administration, Methodology, Funding acquisition, Formal analysis, Conceptualization.
