# Supplementary figures and images for: GABAA receptors and neuroligin 2 synergize to promote synaptic adhesion and inhibitory synaptogenesis
Source: Front Cell Neurosci. 2024 Jul 18;18:1423471. doi: 10.3389/fncel.2024.1423471 (PMC11295144; doi:10.3389/fncel.2024.1423471)

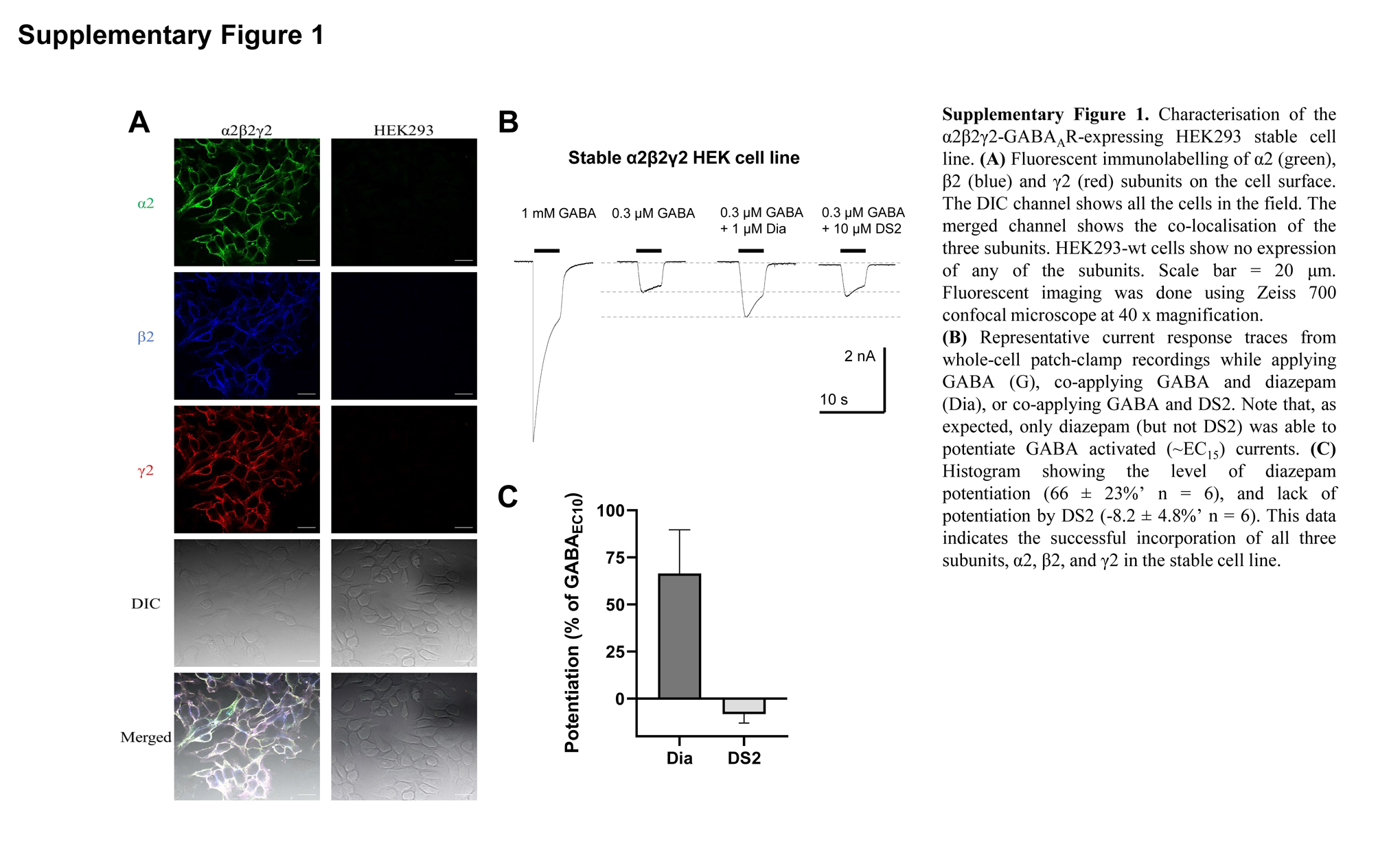

Supplement: Supplementary file 1 [file Image_1.TIF]

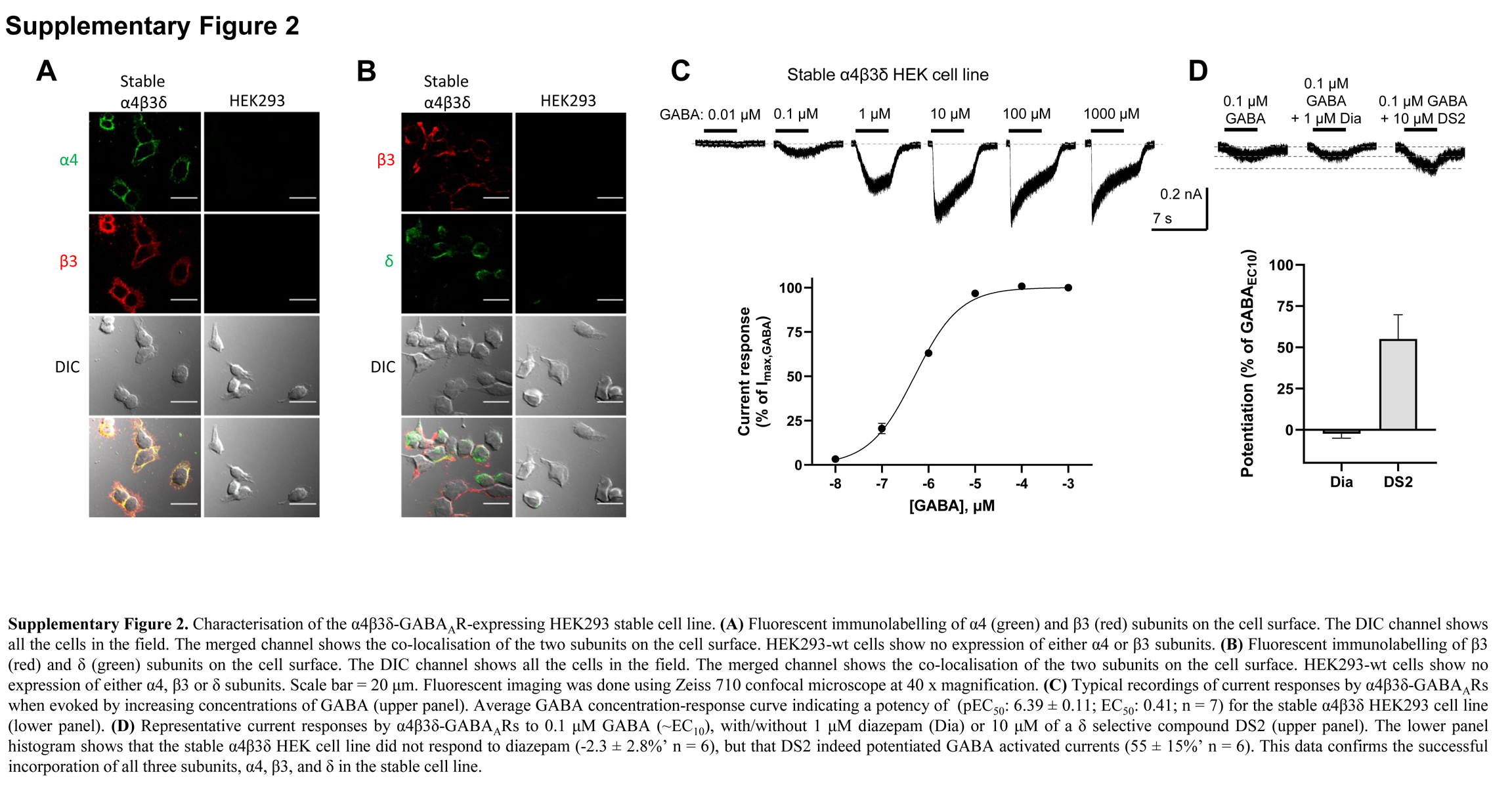

Supplement: Supplementary file 2 [file Image_2.TIF]

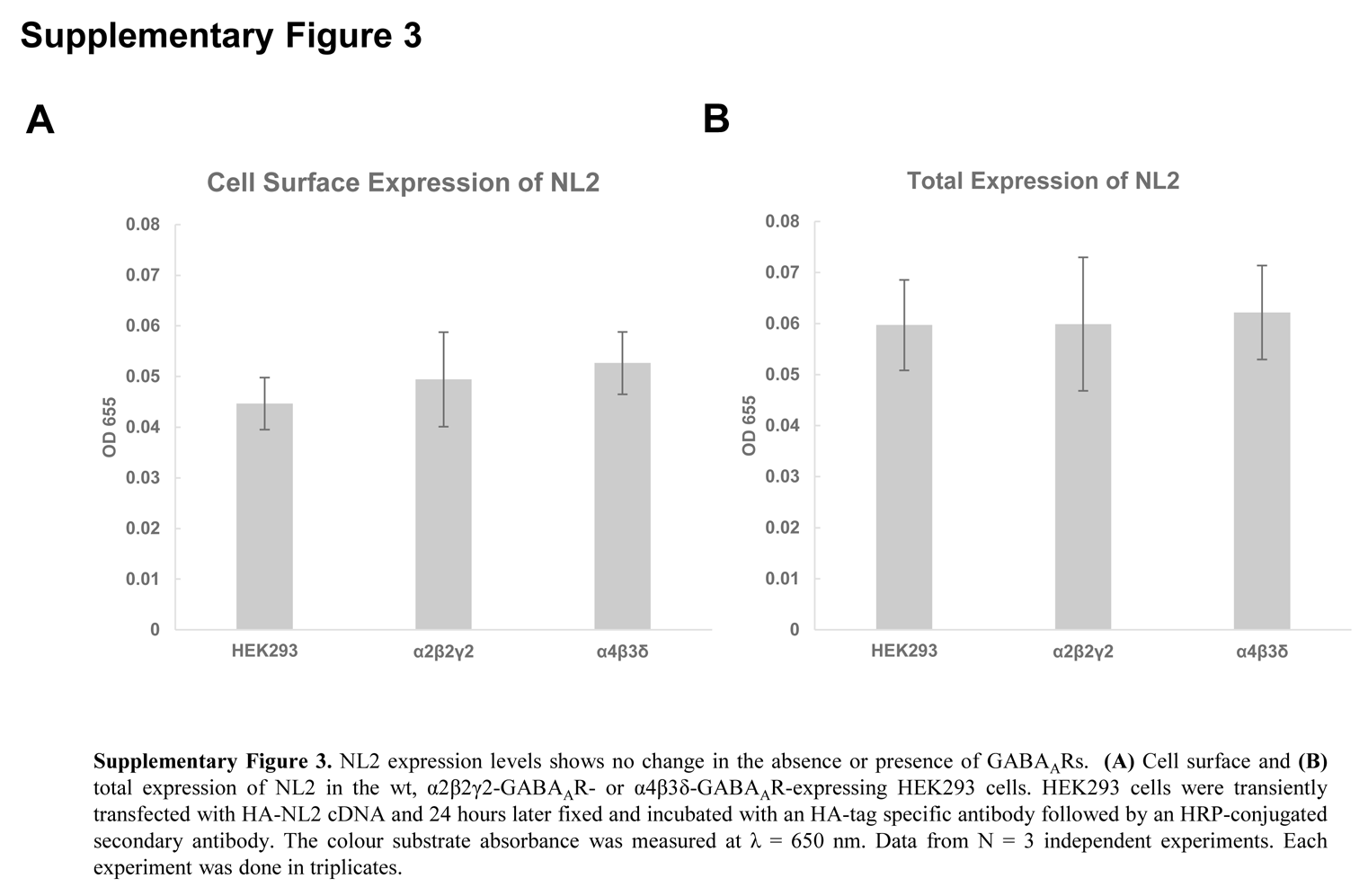

Supplement: Supplementary file 3 [file Image_3.TIF]

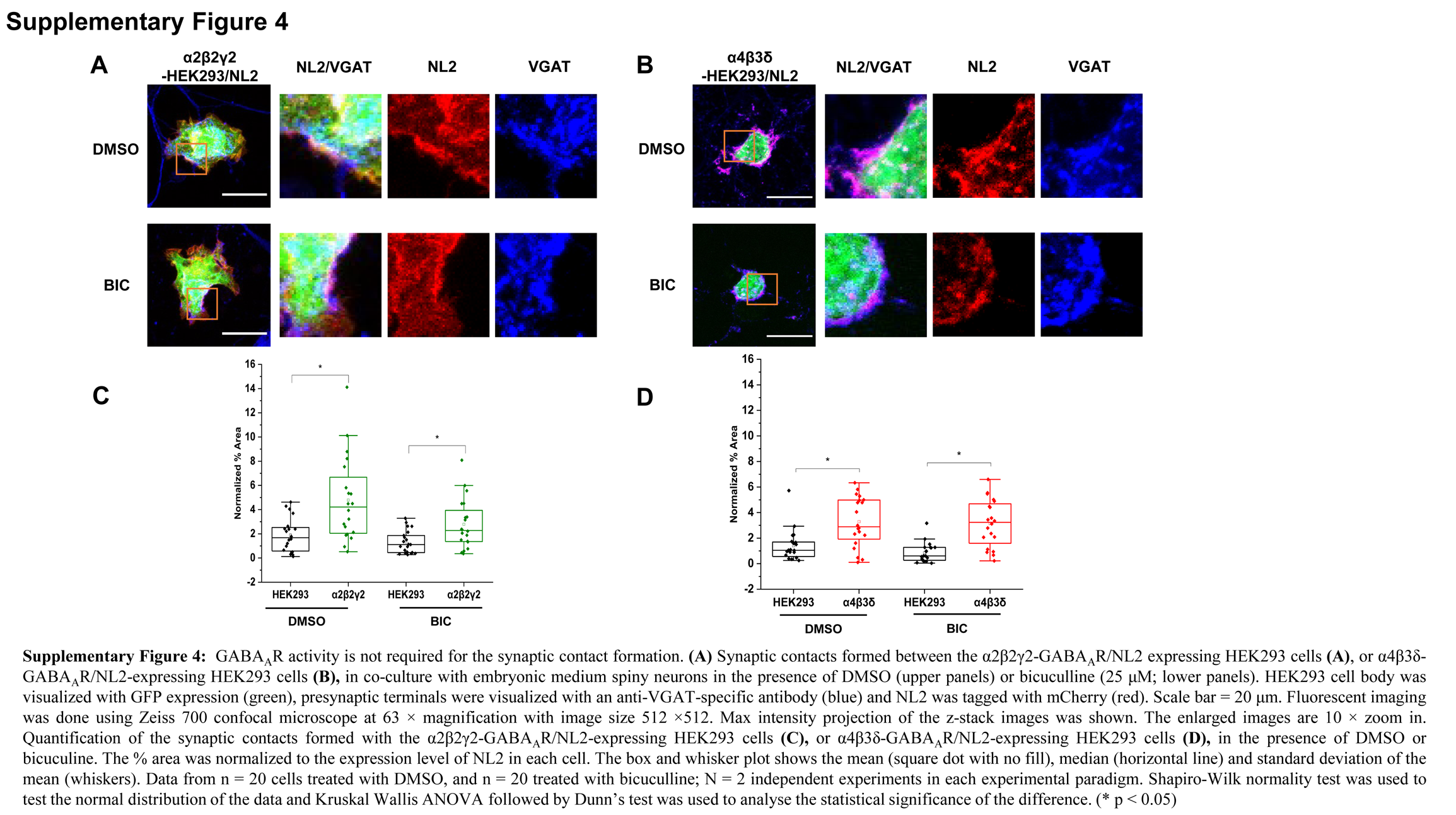

Supplement: Supplementary file 4 [file Image_4.TIF]
